# A Key Motif in the Cholesterol-Dependent Cytolysins Reveals a Large Family of Related Proteins

**DOI:** 10.1128/mBio.02351-20

**Published:** 2020-09-29

**Authors:** Jordan C. Evans, Bronte A. Johnstone, Sara L. Lawrence, Craig J. Morton, Michelle P. Christie, Michael W. Parker, Rodney K. Tweten

**Affiliations:** a Department of Microbiology and Immunology, University of Oklahoma Health Sciences Center, Oklahoma City, Oklahoma, USA; b Department of Biochemistry and Molecular Biology, Bio21 Molecular Science and Biotechnology Institute, University of Melbourne, Parkville, Victoria, Australia; c St. Vincent’s Institute of Medical Research, Fitzroy, Victoria, Australia; University of Pittsburgh School of Medicine

**Keywords:** *Bacteroides*, *Bacteroidetes*, *Chryseobacterium*, *Deinococcus*, *Elizabethkingia*, MACPF, *Thalassiosira oceanica*, pore-forming, toxin

## Abstract

The cholesterol-dependent cytolysins (CDCs) are bacterial, β-barrel, pore-forming toxins. A central enigma of the pore-forming mechanism is how completion of the prepore is sensed to initiate its conversion to the pore. We identified a motif that is conserved between the CDCs and a diverse family of nearly 300 uncharacterized proteins present in over 220 species that span at least 10 bacterial and 2 eukaryotic phyla. Except for this motif, these proteins exhibit little similarity to the CDCs at the primary structure level. Studies herein show this motif is a critical component of the sensor that initiates the prepore-to-pore transition in the CDCs. We further show by crystallography, single particle analysis, and biochemical studies of one of these CDC-like (CDCL) proteins from Elizabethkingia anophelis, a commensal of the malarial mosquito midgut, that a high degree of structural similarity exists between the CDC and CDCL monomer structures and both form large oligomeric pore complexes. Furthermore, the conserved motif in the *E. anophelis* CDCL crystal structure occupies a nearly identical position and makes similar contacts to those observed in the structure of the archetype CDC, perfringolysin O (PFO). This suggests a common function in the CDCs and CDCLs and may explain why only this motif is conserved in the CDCLs. Hence, these studies identify a critical component of the sensor involved in initiating the prepore-to-pore transition in the CDCs, which is conserved in a large and diverse group of distant relatives of the CDCs.

## Abstract (precis)

The cholesterol-dependent cytolysins’ pore-forming mechanism relies on the ability to sense the completion of the oligomeric prepore structure and initiate the insertion of the β-barrel pore from the assembled prepore structure. These studies show that a conserved motif is an important component of the sensor that triggers the prepore-to-pore transition and that it is conserved in a large family of previously unidentified CDC-like proteins, the genes for which are present in a vast array of microbial species that span most terrestrial environments, as well as most animal and human microbiomes. These studies establish the foundation for future investigations that will probe the contribution of this large family of CDC-like proteins to microbial survival and human disease.

## INTRODUCTION

**FIG 1:**
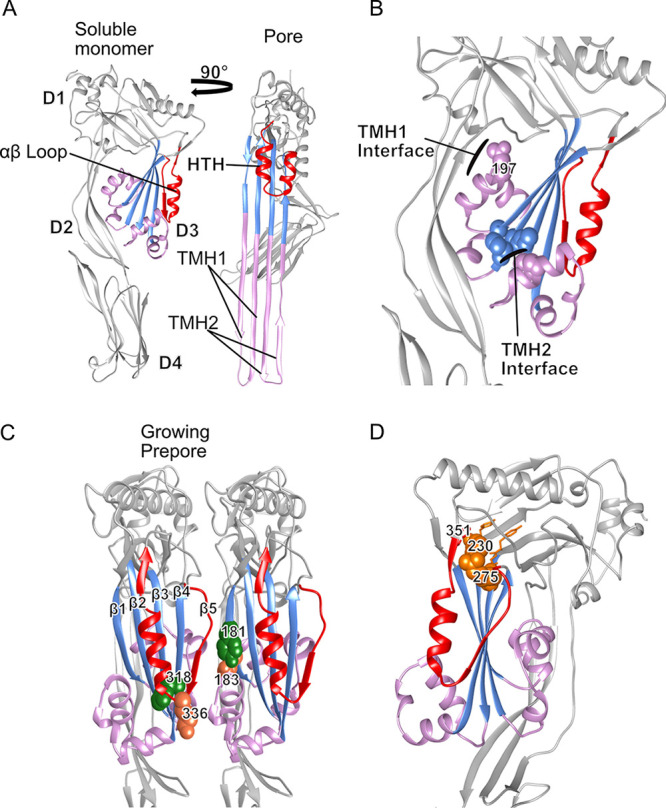
PFO structure showing critical residues for the mechanism of pore formation and the conserved CDC domain 3 motif. (A) The PFO crystal structure showing the core β-sheet (light blue) and the flanking αHBs (pink) in the soluble monomer (left) which unfurl into transmembrane hairpins (TMH) in the pore model (right). The αβ loop (red) in the soluble monomer refolds into an HTH upon conversion of the prepore to the pore. (B) Side view of PFO emphasizing the two interfaces which must be disrupted for the formation of TMHs and pore conversion. In TMH1, a water network is maintained around N197 (spheres) which stabilizes the interface. The TMH2 interface is maintained by the interaction of two methionine residues on the core β-sheet with a phenylalanine (spheres) in the αHB. (C) Representation of a growing PFO prepore following membrane binding. Following membrane binding, β5 of the αβ loop must be displaced to allow interactions to form between β1 and β4 of two monomers. Movement of β5 also increases the solvent accessibility of label-site residue V322 (green spheres). Upon completion of the prepore, K336 (coral spheres), located on the tip of the αβ loop, forms an electrostatic interaction with E183 (coral spheres) of the neighboring monomer. This interaction is necessary to supply the force to complete the flattening of the core β-sheet and to disrupt the water network around N197, resulting in pore conversion. (D) The conserved CDC D3 motif in the PFO soluble monomer. The conserved motif (orange spheres and sticks) resides on the severe bend in the core β-sheet on strands 2 and 3. R275 makes contacts with the beginning of the αβ loop at the turn between β-strands 4 and 5, and F230 forms a π-stacking interaction with a well-conserved aromatic, F351 (red sticks), at the end of the α-helix.

The cholesterol-dependent cytolysin (CDCs) are a family of β-barrel pore-forming toxins expressed by many Gram-positive pathogens, but only identified in a few Gram-negative terrestrial bacteria to date (1). The CDCs share a domain 3 (D3) protein fold with many evolutionarily distant proteins, including members of the mammalian membrane attack complex/perforin family (MACPF) (2–7). This structural domain is characterized by a core β-sheet flanked by two contiguous α-helical bundles (αHBs) (Fig. 1, Fig. S1 in the Supplemental Material). Membrane-bound CDC monomers self-associate into toroidal, multimeric prepore complexes of 35 to 40 monomers, which undergo large structural rearrangements in order to form the membrane-spanning β-barrel pore. Prior studies in our lab have shown that the conversion of the prepore to pore requires the refolding of the two D3 αHBs into membrane-spanning β-hairpins (TMHs) with an ∼40Å vertical collapse of the complex to form the membrane-spanning β-barrel pore (8–13). The mechanism(s) and structures that initiate the prepore-to-pore transition remain incompletely understood, yet they are at the core of the CDC pore-forming mechanism.

10.1128/mBio.02351-20.3 FIG S1 CDC/MACPF/STNX crystal structures showing the α-helical bundles and αβ loop analogs. The crystal structures of CDCs PFO (1PFO), ALO (3CQF), LLO (4CDB), SLO (4HSC), PLY (5CR6), DLY (6NAL), MACPFs, perforin (3NSJ), complement C9 (6CXO), complement C6 (3T5O), Pleurotolysin B (4OEJ), Photorhabdus luminescens (2QP2), MPEG1/perforin 2 (6U2W), and stonefish proteins stonustoxin α and β subunits (4WVM) are shown with the analogs to the αβ loop (red) and α-helical bundles 1 and 2 (cyan). The regions corresponding to the αβ loop were not visible in the structures for complement C5 (4A5W) and C8α (2QQH and 2RD7), which are not shown. Also, it is unknown if C5 and C8α convert and insert the analogous α-helical bundles as transmembrane β-hairpins into the bilayer. All structures were rendered using UCSF Chimera (E. F. Pettersen, T. D. Goddard, C. C. Huang, G. S. Couch, D. M. Greenblatt, E. C. Meng, T. E. Ferrin. 2004, 25:1605-12, J. Comp. Chem, https://doi.org/10.1002/jcc.20084). Download FIG S1, TIF file, 2.6 MB. Copyright © 2020 Evans et al. 2020 Evans et al. This content is distributed under the terms of the Creative Commons Attribution 4.0 International license.

We have identified a motif, F/Y-F/Y-X_n_-YGR, that is conserved between the CDCs and a large group of uncharacterized proteins (termed herein as CDC-like or CDCL) that exhibit little similarity in their primary structures with the CDCs. The conspicuous presence of this motif in both families of proteins prompted us to investigate its role in the CDC pore-forming mechanism. We show this motif makes critical contacts with an αβ loop in the soluble monomer of the CDC protein perfringolysin O (PFO) (Fig. 1D), which reveals that the αβ loop plays a central role in regulating the transition of the prepore to the pore. Importantly, the crystal structure for one CDCL from Elizabethkingia anophelis reveals that it exhibits a high degree of structural similarity with domains 1 to 3 (D1 to D3) of the CDCs and that the residues of the conserved F/Y-F/Y-X_n_-YGR motif occupy nearly identical positions and make similar contacts in the *E. anophelis* CDCL crystal structure to those in PFO. These studies reveal the critical nature of the F/Y-F/Y-X_n_-YGR motif to the CDC pore-forming mechanism and provide the first characterization of a representative of the CDCL family of proteins.

## RESULTS

### Primary structures of a large class of uncharacterized proteins share a conserved F/Y-F/Y-X_n_-YGR motif with the CDCs.

The alignment of ∼300 CDCL and over 100 CDC primary structures revealed a conserved motif consisting of F/Y-F/Y-X_n_-YGR. An example is shown in Fig. S2, where the predicted primary structures of selected CDCLs from 12 genera were aligned with PFO. The F/Y-F/Y-X_n_-YGR motif and a downstream glycine pair (Fig. S2) are highly conserved, whereas there are relatively few other conserved residues or conservative substitutions. We have previously shown that the conserved glycine pair in PFO (G324 and G325), which is located at the beginning of the αβ loop in the CDCs, does not tolerate substitution with side chain amino acids and traps it in a prepore complex (14). This alignment also shows there is no similarity between the CDCL domains that correspond to the conserved membrane-binding D4 of the CDCs, Furthermore, the classic signature undecapeptide (ECTGLAWEWWR) and cholesterol binding (TL pair) motifs of the CDC membrane-binding D4 (15, 16) are absent in the CDCLs. The first aromatic residue of the F/Y-F/Y-X_n_-YGR motif was found to vary in these proteins (62% conservation as an aromatic, the rest have S, Q, A, or N), although the rest of the motif residues, including the GG pair, were conserved in 92 to 99% of the CDCLs. The conspicuous conservation of the F/Y-F/Y-X_n_-YGR across the CDC and CDCL primary structures prompted us to investigate its role in the CDC pore-forming mechanism.

10.1128/mBio.02351-20.4 FIG S2 Alignment of selected CDCLs primary structures. The alignment of the primary structures of selected CDCLs from each phylum with that of the CDC PFO from Clostridium perfringens (*Firmicutes*) is shown. The phyla represented are (1) *Balneolaeota*, (2) *Rhodothermaeota*, (3) *Cyanobacteria* (*Cyanophyta*), (4) *Proteobacteria* (δ-proteobacteria), (5) *Eukaryota*, (6) *Bacteroidetes*, (7) *Ignavibacteriae*, (8) *Archeae*, (9) *Armatimonadetes*, (10) *Deinococcus-Thermus*, (11) *Actinobacteria*, and (12) *Spirochaetes*. Except for the asterisked species, all of the primary structures shown are predicted to be lipoproteins. Those species names with asterisks are predicted to be cytoplasmic, although it is possible they are secreted by mechanisms that are currently unknown, as comparatively little or nothing is known about protein secretion in these phyla. Of the *Deinococcus* species that exhibit a putative CDCL, most are predicted to have a type I or II signal peptidase cleavage site, whereas *D. deserti*, shown here, does not have a predicted signal peptidase I or II cleavage site. Most of the species from the *Bacteroidetes* phylum exhibit predicted lipoprotein structures and signal peptidase II cleavage sites. In addition to the conservation of the F/Y-X_n_-YGR motif and the GG motif, we also see that residues which are equivalent to PFO residues Q228, P359, and G360 are highly conserved. The reason for the conservation of these residues in the CDCs and the CDCLs is unclear. Altering Q228 to alanine in PFO does not significantly alter pore-forming activity (data not shown). The PG pair (PFO residues P104 and G105) is buried in D1 in the CDCs, which suggests a structural role. Those residues shown to be highly conserved have the equivalent PFO residue numbers above them. The alignment was carried out using MUSCLE using the default parameters (R. C. Edgar, *BMC Bioinformatics* 5:113, 2004, https://doi.org/10.1186/1471-2105-5-113; R. C. Edgar, *Nucleic acids research* 32:1792–1797, 2004, https://doi.org/10.1093/nar/gkh340). Download FIG S2, DOCX file, 0.03 MB. Copyright © 2020 Evans et al. 2020 Evans et al. This content is distributed under the terms of the Creative Commons Attribution 4.0 International license.

### Pore formation by PFO mutants with alterations in the conserved F/Y-F/Y-X_n_-YGR CDC motif.

The conserved F/Y-F/Y-X_n_-YGR motif in PFO, the archetypical CDC, is F^230^-Y-X_41_-Y^273^GR and is located at the bends of β-strands 2 and 3 between domains 3 and 1 (D3 and D1) (Fig. 1D). When individually mutated to alanine, we generally observed greater than wild-type activity accompanying a 1 to 3°C decrease in melting temperature (*T_m_*) of PFO, with the exception being R275A, which exhibits less than 1% of wild-type PFO activity (Table 1). Therefore, we initiated our investigation of this conserved motif to first understand the basis of losing pore-forming activity in the R275A mutant.

**TABLE 1:** Relative pore-forming activities and *T_m_* values for PFO and its derivatives^*a*^

Strain	%WT EC_50_	*T_m_* (°C [ΔWT PFO])^*b*^
PFO	100	49.5
F230A	95–160	48 (−1.5)
F230C	110	48.3 (−1.2)
F230C:NBD	79	ND
F230L	140	46.3 (−3.2)
Y231A	320	44.5 (−5.0)
Y273A	350	44.4 (−5.1)
G274A	70–120	40.3 (−9.2)
R275A	<1	46.5 (−3.0)
R275K	10	46.7 (−2.8)
D326A	80	ND
F351A	150	48.6 (−0.9)
F351C	110	48.5 (−1.0)
F351C:NBD	50	ND
N197W:R275A	100	39.5 (−10)
F230A:F351A	5	46.3 (−3.2)
F230C:F351C (ox)	3	46.6 (−2.9)
F230C:F351C (red)	430	45.7 (−3.8)
F230L:F351L	250	47.1 (−2.4)
Y231A:Y273A	295	37.4 (−12.1)
T319C:V334C (ox)	<1	ND
T319C:V334C (red)	100	48.4 (−1.1)
N197W:F230A:F351A	210	41.0 (−8.5)
N197W:F230C:F351C (ox)	25 (6)^*c*^	41.9 (−7.6)
N197W:F230C:F351C (red)	510	41.8 (−7.7)
F230C:F351C:V322C (ox)	12 (3)^*c*^	46.3 (−3.2)
F230C:F351C:V322C (red)	85 (20)^*c*^	46.0 (−3.5)
F230C:F351C:V322C-NBD (ox)	<1	ND
F230C:F351C:V322C-NBD (red)	<1	ND
R275A:T319C:V334C (ox)	<1	49.5 (0.0)
R275A:T319C:V334C (red)	<1	47.7 (−1.8)

a The pore-forming activity for each mutant relative to the pore-forming activity of wild-type PFO was determined from the 50% effective concentration (EC_50_) of pore formation on carboxyfluorescein-loaded, cholesterol-rich liposomes and reported as the percentage of PFO activity at 37°C (% activity = [EC_50_ PFO/EC_50_ mutant] × 100). Reduced (red) samples contained 5 mM DTT, while oxidized (ox) samples contained no reducing agent. The results are representative of 3 or more experiments.

b ND, the *T_m_* for the NBD-labeled derivatives could not be determined since the necessary concentrations could not be achieved for the analysis.

c The values in parentheses reflect the percentage of pore-forming activity of these mutants in their oxidized state compared to the activity in the prereduced PFO^F230C:F351C^, which was 510% of wild-type PFO.

### R275 forms interactions with the αβ loop critical to oligomer geometry and pore formation.

R275 forms its strongest interactions via side-chain-mediated interactions with the backbone and side chain of N348, the backbone of G325, and a charge interaction with D326, which are located at the N-terminal (N348) and C-terminal (G325 and D326) ends of the αβ loop (Table S1). The PFO^D326A^ pore-forming activity was 80% of wild-type PFO, whereas PFO^R275K^ exhibited only 10% of wild-type activity, suggesting that the side-chain-mediated backbone interactions of R275 with N348 and G325, and not its charge, are the primary stabilizing interactions for the two ends of the αβ loop. These interactions are lost in the R275A mutant (Table S1), which is likely responsible for the inactive phenotype of this mutant. Therefore, we investigated why these R275-mediated interactions with the two ends of the αβ loop are necessary to form the pore.

10.1128/mBio.02351-20.1 TABLE S1 Amino acid interaction energies (IE) of the residues Y275, F230, F351, Y231, Y273, and G274 and their derivatives, as determined by the interaction energy matrix analysis (Pettersen EF et al., J Comput Chem 25:1605-12 https://www.ncbi.nlm.nih.gov/pubmed/18214960). In parentheses are the changes in IE from wild-type PFO. All structures were energy minimized (including wild-type PFO) with Chimera 1.14 (Pettersen EF, Goddard TD, Huang CC, Couch GS, Greenblatt DM, Meng EC, Ferrin TE. 2004. J Comput Chem 25:1605-12 https://www.ncbi.nlm.nih.gov/pubmed/15264254) using the default parameters. The interaction energies were determined between the side chain of the residue listed below versus all other residues of the side chain and backbone (except for the native G274, where backbone versus residue interactive energies were determined). Download Table S1, DOCX file, 0.02 MB. Copyright © 2020 Evans et al. 2020 Evans et al. This content is distributed under the terms of the Creative Commons Attribution 4.0 International license.

**FIG 2:**
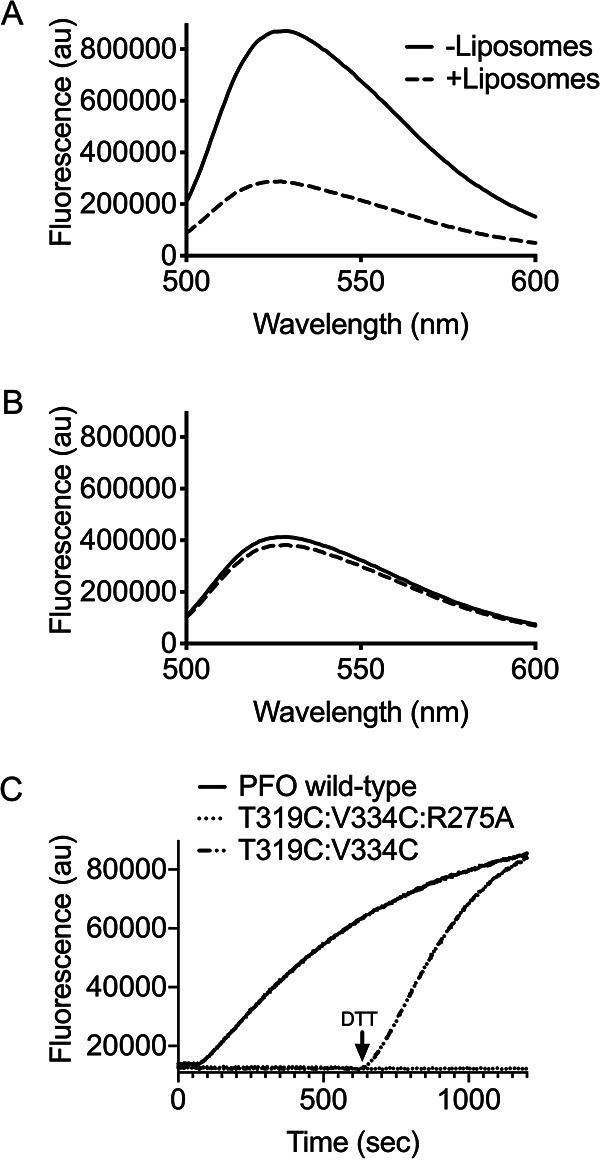
Residue R275 is necessary for αβ loop stability and conversion of the prepore to the pore. (A) Fluorescence scanning of NBD-labeled PFO^V322C^ shows β5 movement away from β4 upon membrane binding, as indicated by a decrease in fluorescence emission (14). Decrease in NBD fluorescence was due to an increased interaction with a polar solvent, i.e., water. (B) Fluorescence scanning of NBD-labeled PFO^R275A:V322C^ revealed β5 movement in the absence of liposomes, as indicated by the low fluorescence emission for both samples. (C) Kinetics of PFO mutants (5 μg) injected into HBS containing carboxyfluorescein-loaded liposomes. PFO wild type (WT) formed pores rapidly on liposomes, allowing release of entrapped dye. PFO^T319C:V334C^, which forms a disulfide bond between β4 and β5, was allowed to form prepores prior to reduction of the disulfide bond with DTT, which led to rapid pore formation. PFO^T319C:V334C:R275A^ was allowed to form prepores, but upon reduction of the disulfide bond, pore formation could not be detected.

**FIG 3:**
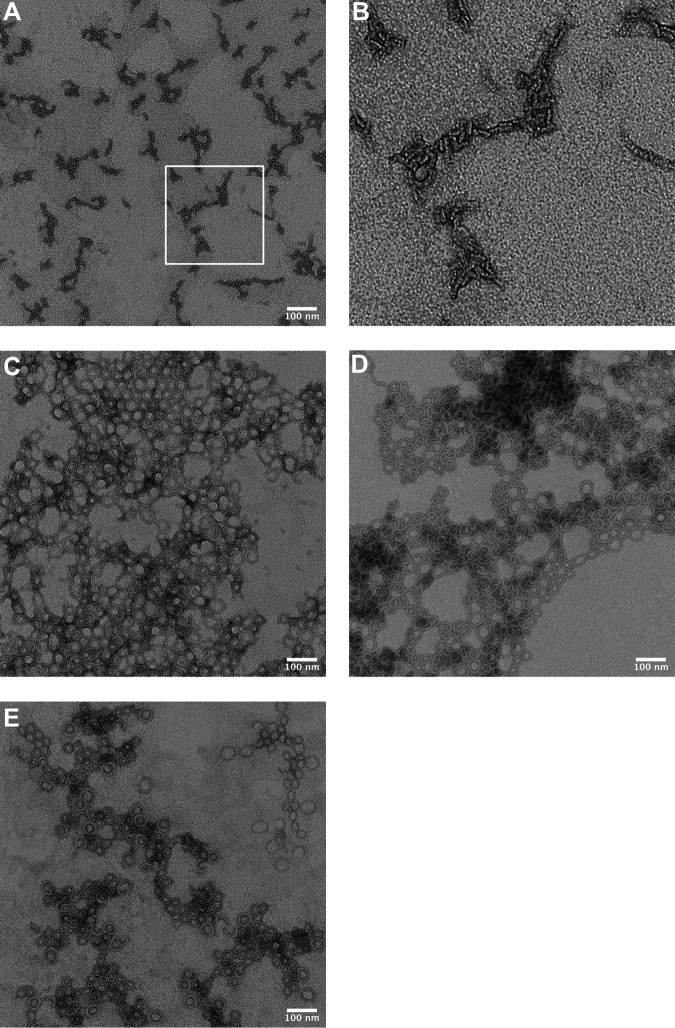
TEM images of membrane-bound oligomers of PFO^R275A^ and derivatives. (A) PFO^R275A^ incubated with cholesterol-containing liposomes prior to staining and imaging reveals linear oligomer complexes. (B) The boxed section shown in panel (A) digitally magnified 10×. (C) Oligomers of PFO^R275A:N197W^. (D and E) Images of PFO^R275A^ oligomers in which an engineered disulfide bond between β-strands 4 and 5 was generated by substituting cysteines for T319 and V334, as previously described (14) under oxidizing (D) and reducing (E) environments. Only PFO^R275A:N197W^ exhibits pore-forming activity (Table 1).

Strand β5 of the αβ loop is displaced from β4 to allow formation of intermolecular backbone hydrogen bonds between β4 and β1 of two monomers during the assembly of the pore (Fig. 1C) (14, 17). The environmentally sensitive probe NBD, when covalently bound to cysteine-substituted V322 on β4 undergoes a nonpolar to polar transition, as β5 disengages from β4 and the αβ loop swings away from the monomer-monomer interface (13, 14). The fluorescence emission of PFO^R275A:V322C-NBD^ in the presence and absence of cholesterol-rich liposomes revealed that the αβ loop was already displaced in the soluble PFO^R275A^ compared to PFO (Fig. 2A and B) (14). When PFO^R275A^ was analyzed by SDS agarose gel electrophoresis (SDS-AGE), no oligomers were detected, but transmission electron microscopy (TEM) revealed the presence of linear oligomers (Fig. 3A and B). To prevent β5 displacement in PFO^R275A^, a disulfide bond was engineered between β4 and β5, as previously described (14). Oxidized PFO^T319C:V334C^ is inactive, but activity is restored whether the disulfide bond is reduced before or after the prepore is assembled (14). When PFO^R275A:T319C:V334C^ is reduced before or after prepore assembly on liposomes, it remains inactive (Fig. 2C); however, the typical circular geometry of the CDC oligomers is restored (Fig. 3D and E). Hence, the premature displacement of this loop apparently results in conformational changes within the PFO structure that prevent the appropriate monomer-monomer contacts necessary for forming a circular complex. However, this does not explain why stabilizing the interaction between β4 and β5 and restoring the formation of circular oligomers does not restore pore-forming activity, which is explored in the next section.

### E183:K336 electrostatic interaction requires R275-mediated interactions with the αβ loop.

The sequential formation of intermolecular π-stacking and electrostatic interactions drive the flattening of the core β-sheet (18) by bringing β-strands 1 and 4 of two adjacent monomers into alignment within the prepore complex to form the upper wall of the β-barrel pore (13, 17). Loss of either intermolecular interaction traps PFO in a prepore complex. Activity can be specifically restored to mutants lacking the electrostatic interaction by the substitution of N197 with tryptophan (19), which disrupts the water network that stabilizes the interface between αHB1 and D1 and D2 (Fig. 1B), thereby allowing the αHBs to refold into the TMHs to form the β-barrel pore (Fig. 1A) (8, 9, 20). Pore-forming activity and circular oligomer formation was restored in PFO^R275A:N197W^ (Table 1, Fig. 3C). K336 of the electrostatic pair resides at the tip of the αβ loop. These results reveal that R275-mediated interactions with the αβ loop are necessary to the formation of a functional electrostatic interaction that drives the prepore-to-pore transition (19).

### Impact of the F230:F351 π-stacking interaction on PFO pore-forming activity.

PFO residue F230 of the conserved motif forms a parallel, offset π-stacking interaction with a well-conserved aromatic at the C-terminal end of the αβ loop (F351 in PFO) (Fig. 1D). The individual substitution of alanine for either F230 or F351 (PFO^F230A:F351A^) reduced activity to 5% of wild-type PFO, whereas the substitution of both with leucine increased activity up to 250% of that for wild type (Table 1). The change in the interaction energy (21) for PFO and its derivatives after energy minimization of the structures (22) is shown in Table S1. The attractive interaction energy between residues 230 and 351 for the active mutants fell between the values for wild type (strongest) and the inactive F230A:F351A mutant (weakest). Hence, weaker interactions between residues 230 and 351 increased activity, up to a point, beyond which activity was largely lost (i.e., F230A:F351A). The F230A mutant was unusual in that it exhibited variable activity, which suggests that a fraction did not maintain the necessary interaction with F351 to function. We show that the loss of activity in the F230A:F351A mutant results from the inability to form a functional intermolecular electrostatic interaction, as the introduction of the N197W mutation rescued pore-forming activity of PFO^F230A:F351A^ (Table 1). Therefore, the F230:F351 π-stacking interaction and the R275-mediated interactions together are necessary to anchor the two ends of the αβ loop, which is required for a functional intermolecular electrostatic interaction.

### Flexibility of the F230-F351 interaction is required for pore formation.

**FIG 4:**
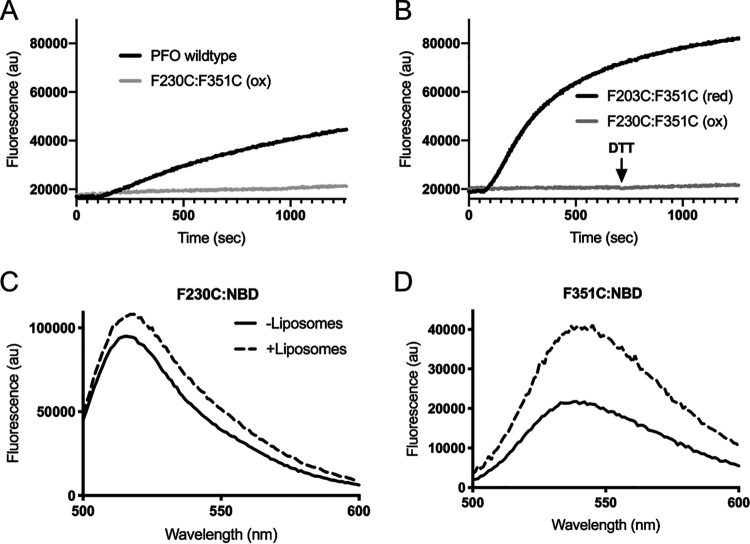
The F230 and F351 stacking interaction must remain flexible during pore formation. (A) Kinetics of PFO mutants (250 ng) injected into HBS containing CF-loaded liposomes. PFO^F230C:F351C^ in its disulfide cross-linked form (ox) had no detectible activity relative to PFO WT. (B) Oxidized PFO^F230C:F351C^ (ox) was incubated with liposomes and allowed to form prepores prior to the addition of DTT (injected at 750 s) and displayed no detectible activity. Reduction to PFO^F230C:F351C^ (red) prior to addition to liposomes displayed activity greater than that for wild-type PFO. (C) PFO^F230C^ labeled with NBD was incubated in the absence (solid line) and presence (broken line) of liposomes representing the soluble monomer and membrane-bound pore complex, respectively. A very minor increase in fluorescence intensity was observed upon pore formation, indicating a shift to a slightly more nonpolar environment in the pore. (D) PFO^F351C^ labeled with NBD reveals a 2-fold increase in fluorescence intensity, indicating a change to a more nonpolar environment. Data are representative of a minimum of three replicates. The difference in fluorescence intensity maximum for (C) and (D) are due to differences in labeling efficiency between the two cysteine mutants.

An F230C:F351C mutant was generated wherein >90% of the cysteines formed disulfide bonds. Pore-forming activity is eliminated by the unreduced disulfide bond, whereas its reduction increases activity to >400% of PFO (Fig. 1A, Table 1). Reduced cysteines can form noncovalent interactions within proteins (23), which is consistent with our above observations that weakening the interaction increases the specific activity of pore formation. In contrast to PFO^F230A:F351A^, the oxidized (inactive) and reduced (active) forms of PFO^F230C:F351C^ assemble SDS-resistant and heat-stable circular oligomers on membranes (Fig. S3). Although reduced PFO^F230C:F351C^ exhibits a significant increase in the rate of pore formation if it is reduced after the prepore is formed on liposomes, no activity is recovered (Fig. 4B). Furthermore, addition of the N197W mutation does not significantly restore activity to oxidized PFO^F230C:F351C^ (Table 1). Hence, the oxidized PFO^F230C:F351C^ complex is trapped in a stable, off-pathway prepore, suggesting that β5 remains sterically trapped at the monomer-monomer interface of the prepore unless the disulfide bond is reduced prior to prepore assembly.

10.1128/mBio.02351-20.5 FIG S3 Oligomer formation and stability of the F230 and F351 mutants. (Left) SDS-AGE analysis of SDS-affected and heat-stable oligomer formation by PFO and its derivatives (10μg each). Each protein was mixed with cholesterol-rich liposomes for 30 min and then mixed with SDS sample buffer with or without being heated to 95°C for 3 minutes (as indicated). Disulfide bond-forming mutants were subjected to the same conditions except that they either remained oxidized (ox) or were reduced (red) with 5mM DTT. (Right) FRET experiment that reveals donor (D) and acceptor (A) fluorescently labeled monomers of the F230A:F351A mutant do interact, since they do not form SDS-resistant oligomers, as shown in the left panel. We have shown previously that mutants that do not form SDS-resistant oligomers by SDS-AGE do interact in an SDS-sensitive manner. Using a nondestructive method such as FRET, we show in the right panel that when donor fluorophore-labeled PFO^F230A:F351A^ is mixed with unlabeled PFO^F230A:F351A^ at a 1:4 ratio and added to liposomes, the emission remains unquenched. When the unlabeled PFO^F230A:F351A^ is replaced with acceptor-labeled PFO^F230A:F351A^, we observe approximately an 80% reduction in the donor emission because the monomers oligomerize on the membrane into SDS-sensitive oligomers, similar to what is observed with native PFO and other inactive derivatives that form SDS-sensitive oligomers (R. Ramachandran, R. K. Tweten and A. E. Johnson. *P Natl Acad Sci USA* 102:7139–7144, 2005, https://doi.org/10.1038/nsmb793; E. M. Hotze, A. P. Heuck, D. M. Czakowsky, Z. Shao, A. E. Johnson, R. K. Tweten, *J Biol Chem* 277:11597–11605, 2002 https://doi.org/10.1074/jbc.M111039200; Hotze et al. *J Biol Chem* 287:24534–24543, 2012, https://doi.org/10.1074/jbc.M112.380139). Download FIG S3, TIF file, 0.6 MB. Copyright © 2020 Evans et al. 2020 Evans et al. This content is distributed under the terms of the Creative Commons Attribution 4.0 International license.

The necessary flexibility of this interaction was further demonstrated by covalently modifying the sulfhydryls of active F230C and F351C single mutants with the environmentally sensitive NBD (9) probe and following its emission as each bound to and formed pores on cholesterol-rich liposomes (Fig. 4C and D, respectively). In both cases emissions increased, indicating that the polarity of their environments decreased, which suggests their relative positions change between the soluble monomer and pore states.

### Displacement of the αβ loop in soluble PFO monomers of active mutants prevents pore formation.

**FIG 5:**
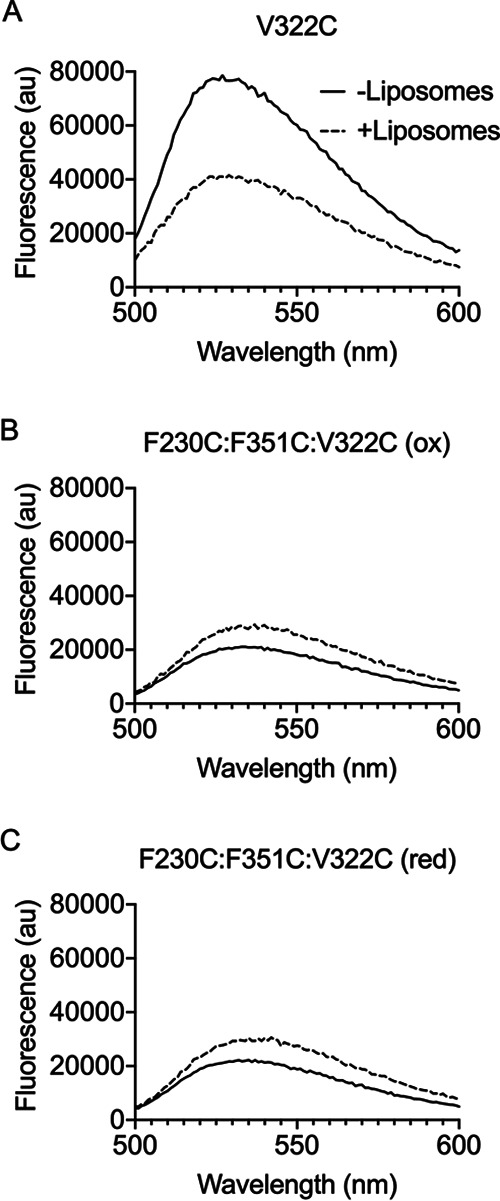
Displacement of the α1β5 loop in the soluble PFO monomer blocks pore formation. PFO^V322C^ (A) labeled with NBD was incubated in the presence and absence of liposomes followed by fluorescence emission scanning. The decrease in emission profile for the sample incubated in the presence of liposomes (broken line) compared to the absence of liposomes (solid line) indicates β5 has moved away from β4, which is necessary for the HTH and pore formation. PFO^F230C:F351C:V322C^ was labeled in the oxidized form to prevent off-target NBD labeling. (B and C) The emission scans of the NBD-labeled PFO^F230C:F351C:V322C^ were obtained in the presence and absence of liposomes in its oxidized (B) and reduced (C) states. Both forms show decreased emission in samples without liposomes, indicating β5 movement. These data are representative of a minimum of three replicates.

We next determined the effect of displacing the αβ loop in an active mutant. When oxidized PFO^F230C:F351C:V322C^ is labeled at V322C, the emission scans of the soluble monomer reveal the αβ loop is displaced in its oxidized and reduced forms (Fig. 5). The NBD labeling of V322C in PFO^F230C:F3251C^ likely displaced the αβ loop, since reduced PFO^F230C:F351C^ is ∼4 times as active as wild-type PFO, whereas NBD-labeled V322C does not displace the αβ loop or eliminate activity in wild-type PFO (14). Hence, the premature displacement of the αβ loop prevents the prepore-to-pore transition.

### Interactions mediated by Y231, Y273, and G274 stabilize D3.

Y231, Y273, and G274 of the motif do not interact with the αβ loop, yet alanine substitution of Y231 and Y273 increased the specific activity by 300 to 350% over wild-type PFO, whereas the alanine mutant of G274 resulted in an unstable protein that caused pore-forming activity to exhibit variation between assays (Table 1). Y231 and Y273 form a π-stacking interaction at the bend between β2 and β3 (Fig. 1D) and G274 resides at the bend in β3. In sharp contrast to the small changes in melting temperatures (*T_m_*) determined for the various mutants of F275 and F230 (−1 to 3°C), the individual and double alanine mutants of Y231 and Y273 and the mutants of G274 decreased their melting temperatures by −5 to −12°C (Table 1). The largest changes in *T_m_* were observed for G274A (−9°C) and Y231A:Y273A (−12°C). The increased activity and lower *T_m_*s for the mutants of Y231 and Y273 are similar to what we have observed for mutations that destabilize D3, which makes it easier for D3 to unfold and form the β-barrel pore (8, 9, 18, 19). In contrast, alanine-substituted G274 results in an unstable protein with variable activity, which appears to be the result of large side-chain-dependent repulsive forces caused by the alanine sidechain, specifically with the backbones of Y273 and R275 (Table S1).

These studies show the F/Y-F/Y-X_n_-YGR motif of the CDCs exhibits a dual role: (i) F230 and R275 form critical contacts that are important for the αβ loop control of the prepore-to-pore transition; and (ii) Y231, Y273, and G274 stabilize the D3 structure of the soluble monomer. The conservation of this motif between the CDCs and CDCLs initially motivated us to investigate its importance to the CDC pore-mechanism. Therefore, we next determined whether this conservation was coincidental or if the CDCLs were a family of distant CDC relatives.

### Phylogenetic analysis of CDCs and CDCLs.

**FIG 6:**
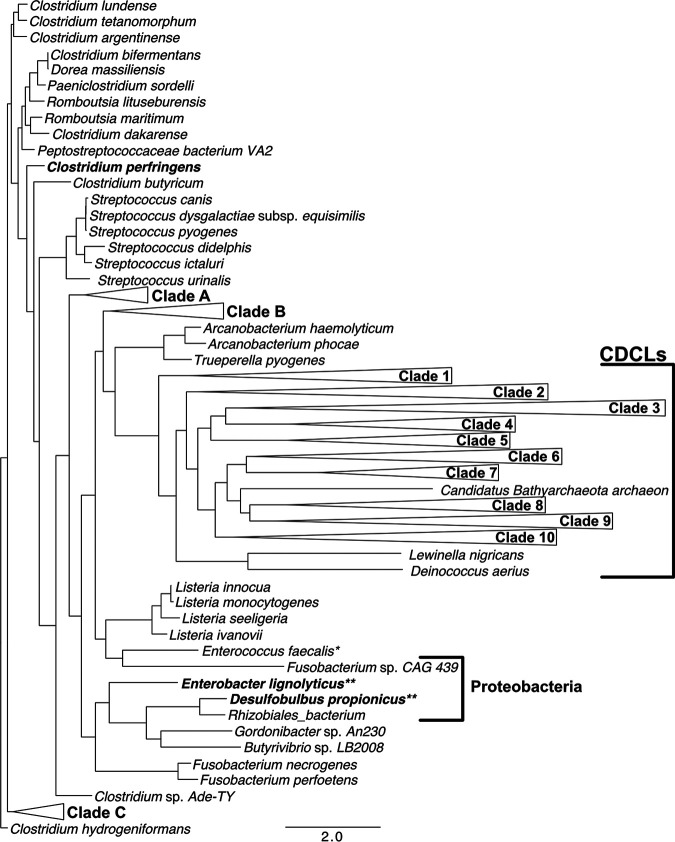
Phylogenetic analysis of the CDCs with the CDCLs. Sequences were first aligned using MUSCLE (41, 53) and then IQ-tree (54–56) was used to perform a maximum likelihood analysis of the aligned primary structures of the current known CDCs and putative CDCLs using a bootstrap value of 200 and the default values set by the software. The tree figure was drawn using FigTree 1.4.4 (57). The single asterisk denotes a strain of Enterococcus faecalis derived from the marine environment, for which clinical isolates have not been identified with a gene encoding either a CDC or a CDCL. The double asterisks denote two Gram-negative species having the cognate D4 structure of the CDCs and shown to bind to and lyse cholesterol-containing membranes (1). The species within each clade are listed in Fig. S4 and each clade structure is shown.

A maximum likelihood phylogenetic analysis of the CDCs and putative CDCLs is shown in Fig. 6. The CDCLs could be divided into several major clades, the species of which are shown in Fig. S4. The F/Y-F/Y-X_n_-YGR motif is also present in a smaller number of CDCLs that lack the C-terminal region of the CDCLs (Fig. S5). These short CDCLs (CDCL^S^) are most often present in an operon with a full-length CDCL, as shown for Elizabethkingia anophelis strain AG1 (Fig. S6) and are most frequently present in species from the *Bacteroides*, *Chitinophaga*, *Chryseobacterium*, and *Elizabethkingia* genera. The CDCL^S^ primary structures often lack significant similarity to the cognate CDCL: the *E. anophelis* AG1 CDCL and CDCL^S^ only exhibit 36% identity (Fig. S6). Most of the CDCLs are produce by species that are not known to be pathogens, although several are produced by bacteria known to cause or are associated with disease, which include Bacteroides fragilis, implicated in peritonitis and abscesses, several *Prevotella* that are associated with oral disease, and Flavobacterium columnare and *Tenacibaculum* species, which are known fish pathogens.

10.1128/mBio.02351-20.6 FIG S4 Species that contain genes for putative CDCLs from the clades identified in Fig. 6. The species contained in the clades shown as horizontal triangles in Fig. 6 are individually expanded here to show the species. Clades A to C are larger clades of the CDCs that were collapsed for convenience, whereas clades 1 to 10 contain CDCLs. Those species that carry genes for more than 1 putative CDCL are designated CDCL 1, CDCL 2, etc. Note that not all isolates of a single species may contain a CDCL. For instance, we have identified 16 different Bacteroides fragilis isolates that contain a CDCL, but other isolates do not. Download FIG S4, DOCX file, 2.1 MB. Copyright © 2020 Evans et al. 2020 Evans et al. This content is distributed under the terms of the Creative Commons Attribution 4.0 International license.

10.1128/mBio.02351-20.7 FIG S5 CDCL^S^ phylogenetic analysis. The alignment and maximum likelihood analyses were performed on the CDCL^S^ primary structures, as for the CDCLs in Fig. 6. Download FIG S5, TIF file, 1.1 MB. Copyright © 2020 Evans et al. 2020 Evans et al. This content is distributed under the terms of the Creative Commons Attribution 4.0 International license.

10.1128/mBio.02351-20.8 FIG S6 Operon structure and genes flanking the CDCL and CDCL^S^ in *E. anophelis* strain AG1. Shown is the operon structure and flanking genes from *E. anophelis* AG1 and an alignment of its CDCL and CDCL^S^ primary structures from the genome (P. Kukutla*, et al. PloS one* **9**:e97715, 2014, https://doi.org/10.1371/journal.pone.0097715). The operon structure was extracted from EnsemblBacteria (Howe KL*, et al. Nuc Acids Research* 48:D689–D695, 2020, https://doi.org/10.1093/nar/gkz890). Download FIG S6, TIF file, 1.1 MB. Copyright © 2020 Evans et al. 2020 Evans et al. This content is distributed under the terms of the Creative Commons Attribution 4.0 International license.

### Crystal structure of the CDCL from Elizabethkingia anophelis reveals its relationship to the CDCs.

**FIG 7:**
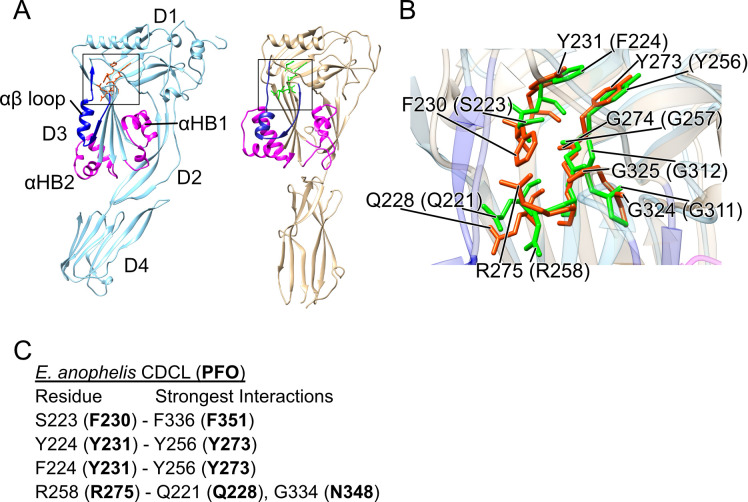
Structure of a CDCL protein from Elizabethkingia anophelis. The recombinant purified *E. anophelis* CDCL expressed and purified from E. coli was crystallized and its structure solved to a 2.09 Å resolution. (A) Ribbon representations of the crystal structure of PFO (1PFO, left) (24) and the *E. anophelis* CDCL (right). For both PFO and the *E. anophelis* CDCL, the αHB1 and 2 that form the membrane-spanning β-hairpins (TMH1 and 2) are shown in magenta and the αβ loop is shown in dark blue. (B) Overlay of the region from both proteins (enclosed in squares in [A]) containing the F/Y-F/Y-X_n_-YGR and GG motifs’ residues and the conserved Q228 of PFO (CDC residues in green and the CDCL residues are in orange). The numbers in parenthesis are the CDCL residues corresponding to those of PFO. (C) Residues of the CDCL that correspond to PFO motif residues F230, Y231, Y273, and R275 and the residues with which they have significant interactions mediated by their side chains compared to those made by the analogous residues in PFO (strongest interactions in bold) determined by their net pairwise interaction energies, as calculated by the interaction energy matrix analysis (21).

The crystal structure of the *E. anophelis* CDCL (Fig. 7A) shows that D1 to D3 are highly similar to the CDC D1 to D3 structure (24) and D3 exhibits the characteristic αHBs of the CDCs, which refold into the TMHs of the β-barrel pore (8, 9). Importantly, the position of the F/Y-F/Y-X_n_-YGR motif residues and the glycine pair are nearly identical to the cognate residues in PFO (Fig. 7B). The structure of the CDCL D4, however, does not resemble the CDC D4 fold and DALI (25) searches with the CDCL D4 structure did not reveal any similar 3D structures. The receptor-binding D4 domains of the CDCs share significant similarity in their primary, secondary, and tertiary structures, and maintain specific signature motifs (15, 16), which are missing in the CDCLs. Furthermore, the D4 domains of the CDCLs exhibit little conservation in their primary structures between genera, and often exhibit little conservation between CDCLs from species that have more than one CDCL gene, as exemplified by 7 full-length CDCLs from *Chitinophaga* sp. strain *MD30* (Fig. S7).

10.1128/mBio.02351-20.9 FIG S7 Alignment of the CDCL primary structures from *Chitinophaga* sp. strain MD30. The same parameters were used as in Fig. S2 to align the primary structures of the CDCLs from *Chitinophaga* sp. MD30. Download FIG S7, DOCX file, 0.02 MB. Copyright © 2020 Evans et al. 2020 Evans et al. This content is distributed under the terms of the Creative Commons Attribution 4.0 International license.

### Pore formation and formation of oligomeric structures by the Elizabethkingia anophelis CDCL and CDCL^S^.

**FIG 8:**
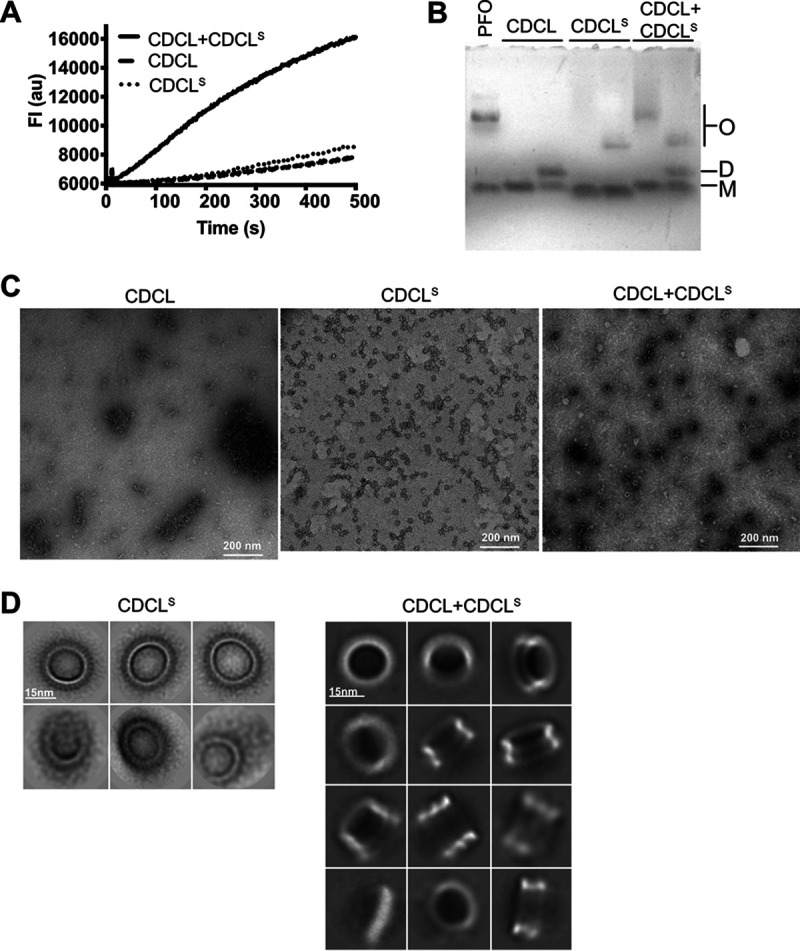
*E. anophelis* CDCL and CDCL^S^ pore formation and oligomeric complexes. (A) Kinetic release of marker (carboxyfluorescein, CF) from cholesterol-rich liposomes when treated with CDCL (1.4 μM), CDCL^S^ (1.9 μM), or CDCL (0.7 μM)+CDCL^S^ (0.95 μM). As CF is released from the liposomes, its emission is dequenched. No change in the emission is observed when the CF liposomes are incubated alone over the time frame of the experiment (not shown). (B) SDS-AGE of oligomer formation by PFO (15 μg), CDCL (15 μg), CDCL^S^ (15 μg), and CDCL^+^CDCL^S^ (15 μg total protein) in the presence of cholesterol-rich liposomes. The first lane of the CDCL, CDCL^S^, and CDCL+CDCL^S^ samples was treated with just SDS sample buffer, whereas the second lane of each sample was treated with sample buffer and heated to 95°C for 3 min. O, oligomer; D, dimer; M, monomer. (C) Negative-stain transmission electron micrographs of cholesterol-rich lipid monolayers treated with CDCL, CDCL^S^, and both (0.05 mg/ml total protein). (D) Selected classes from single-particle analysis of CDCL^S^ alone and in combination with CDCL from raw micrographs as shown in (C).

The addition of both CDCL^S^ and CDCL to carboxyfluorescein-loaded cholesterol-rich liposomes resulted in rapid dye release, which is not observed in the presence of either CDCL^S^ or CDCL alone (Fig. 8A). However, we have to note that although this activity could be observed and replicated on the same day, it could not be repeated with fresh liposomes and the same or freshly purified proteins. We have observed this behavior for other CDCLs and have yet to identify the basis for this behavior. It did not result from contamination with an active CDC, since the individual CDCL and CDCL^S^ proteins at twice the concentration only showed a small release of marker from the liposomes (Fig. 8A). The activity of the combined proteins is 100- to 1,000-fold less than for the CDCs, likely due to the absence of a receptor for the CDCLs on the liposomes, similar to the CD59-binding CDC intermedilysin (26).

The CDCL did not form oligomers on liposomes but appeared to form stable dimers, whereas CDCL^S^ formed SDS-resistant and heat-stable oligomers (Fig. 8B). When the CDCL and CDCL^S^ proteins were combined, they formed an SDS-stable oligomer with a mass slightly higher than that of PFO oligomers (Fig. 8B). When this complex was heated with SDS sample buffer, the size was reduced to that of the CDCL^S^ oligomer. This suggests the CDCL^S^ acts as a scaffold for the assembly of the CDCL, and that the latter’s oligomer is dissociated by SDS when heated. Analysis of the CDCL and CDCL^S^ on lipid monolayers by TEM revealed circular oligomers for the CDCL^S^ but not the CDCL, whereas the oligomeric structures observed in the presence of equimolar concentrations of CDCL^S^ and CDCL appeared different from those of the CDCL^S^ alone (Fig. 8C).

Single-particle analysis (Fig. 8D) revealed that when the CDCL and CDCL^S^ proteins are mixed, stacked oligomers are formed, which is consistent with the observed increase in mass by SDS-AGE when the two proteins are combined. No dual ring structures were observed for the CDCL^S^ alone, suggesting that these stacked oligomers only form when both the CDCL and CDCL^S^ are present. The CDCL^S^ ring contained ∼28 monomers, compared to 35 to 40 observed for CDC oligomers (11, 13). This smaller ring structure would account for the smaller size of the CDCL^S^ complex and the slightly larger size of the CDCL-CDCL^S^ complex compared to that of PFO oligomers (Fig. 7B and D).

### Expression of CDCL and CDCL^s^ in *E. anophelis*.

The *E. anophelis* CDCLs and a large fraction of the putative CDCL proteins from other species exhibit a lipoprotein type II signal peptidase cleavage site and the cognate cysteine. The expression of the CDCL and CDCL^S^ from *E. anophelis* AG1 was followed over time from a culture grown at 31°C (Fig. S8). Affinity-purified rabbit antibody to both proteins was used to probe purified membrane and spent media fractions, which revealed that both proteins are secreted into the spent medium and are first detected in mid-log phase. Much of the CDCL^S^, but not the CDCL, appeared to aggregate into high molecular weight species that did not penetrate into the gel, which is consistent with the above studies, where we observed that the CDCL^S^ proteins oligomerized into SDS-resistant oligomers in the presence of liposomes.

10.1128/mBio.02351-20.10 FIG S8 Expression of CDCLs in *E. anophelis*. (A) The membranes (inner and outer combined) and spent media fractions were isolated at each timepoint (designated by numbers 1 to 10) from a 500-ml culture of *E. anophelis* strain AG1. The membranes were concentrated 60-fold (equivalent to 1.2 ml of culture) and the spent medium was concentrated 30-fold (equivalent to 0.6 ml of culture). (B) Each sample (20 μl) was separated by SDS-PAGE and transferred to nitrocellulose. Each set of blots was probed with affinity-purified antibody generated to purified, recombinant CDCL or CDCL^s^ from E. coli. Note the presence of high mass aggregates (O) in the wells of the Western blot of the CDCL^S^ samples. The immunoreactive material at the top of the gels that is not in the wells of either gel resulted from antibody trapped in gel material stuck to the nitrocellulose membrane. Download FIG S8, TIF file, 2.3 MB. Copyright © 2020 Evans et al. 2020 Evans et al. This content is distributed under the terms of the Creative Commons Attribution 4.0 International license.

## DISCUSSION

Most, if not all, studied β-barrel pore-forming toxins form an obligatory prepore intermediate (10, 27–31), the completion of which must be detected before conversion to the pore; our studies suggest the αβ loop is a critical component of this sensor. The conservation of the F/Y-F/Y-X_n_-YGR motif and little else between the CDCs and the large family of CDCLs provided the basis for these studies that revealed the role of the αβ loop in sensing completion of the prepore and its role in the CDC pore-forming mechanism. These studies show the F/Y-F/Y-X_n_-YGR motif forms critical interactions necessary for the function of the αβ loop. These interactions impact the subsequent formation of the key intermolecular π-stacking and electrostatic interactions, which drive the prepore-to-pore transition (18, 19). Once these two intermolecular interactions are initiated, the prepore is irrevocably committed to its transition to the pore. The residues of the motif play two distinct roles in the CDC mechanism: first, specific residues form interactions with the αβ loop that are critical to its function in regulating the prepore-to-pore transition and second, other residues stabilize the D3 structure, which affects the stability and pore-forming activity of PFO. Subsequent studies into the CDCL structure revealed this motif forms similar contacts with the αβ loop of the CDCLs, which suggests a common role for the CDCLs and reveals the CDCLs to be distant relatives of the CDCs.

In PFO, R275 and the F351:F230 π-stacking interactions anchor the two ends of the αβ loop. If either interaction is eliminated, pore formation is lost. In the R275A mutant, the loss of activity can be directly related to the premature displacement of the αβ loop, which results in the formation of linear oligomers. Although the formation of circular oligomers is restored by locking β5 to β4, pore-formation activity is not restored because the loss of the R275-mediated interactions prevented the subsequent formation of the intermolecular electrostatic interaction, which drives the prepore-to-pore transition (19). When the αβ loop was displaced in the active F230C:F351C mutant, the pore-forming activity was eliminated, likely due to moving K336 (located at the tip of the αβ loop) out of position so it could not form the intermolecular electrostatic interaction with E183 of an adjacent monomer within the prepore oligomer (19). These results were consistent with the observation that the loss of activity in the R275A and F230A:F351A mutants was restored by the introduction of the N197W, which we have shown restores activity in a mutant where the intermolecular electrostatic interaction is lost (19).

The F351:F230 π-stacking interaction of the αβ loop must allow it a certain degree of mobility, as its substitution with a rigid disulfide bond eliminates activity unless it is reduced. Notably, the F351C:F230C mutant is >400% as active as wild-type PFO when the disulfide bond is reduced before adding the monomers to liposomes, whereas if oxidized F230C:F351C is first allowed to assemble the prepore on liposomes, pore formation is not restored by reduction of the disulfide bond. Hence, preventing the movement of the αβ loop at the appropriate times also abrogates pore formation. The results of the studies with the R275 and F230:F351 mutants show that when the ends of the αβ loop are not sufficiently anchored, if it is prematurely displaced or its movement restricted than PFO cannot form a functional intermolecular electrostatic interaction. The strand β5 of the αβ loop undergoes a β-strand to α-helix transition upon pore formation to form the helix-turn-helix (HTH) in the monomers of the pore complex. This transition shortens this loop by about 9 Å, thereby placing additional stress on the K336-E183 electrostatic interaction, which breaks the final contacts between D3 and domains 1 and 2 to allow the formation of the β-barrel pore. Hence, the αβ loop must undergo specific structural transitions in the prepore to facilitate those intermolecular interactions (18, 19) that drive the prepore-to-pore transition.

Residues Y231, Y273, and G274 of this motif, on the other hand, do not interact with the αβ loop but appear to contribute to the stabilization of D3. The elimination of the π-stacking interaction between Y231 and Y273 significantly increased pore-forming activity and the G274A mutant resulted in an unstable pore-forming phenotype. These phenotypes correlated with significant decreases in the melting temperatures of PFO, which is largely dictated by the unfolding of D3 (19). D3 is in a metastable state; it must remain stable in the soluble monomer but undergoes significant secondary and tertiary structural changes to form the β-barrel pore, whereas D1 and D4 remain largely unchanged (8, 9, 11, 13). We have shown that specific intramolecular interactions stabilize the structure of D3 in the monomer, which are broken by the formation of the intermolecular electrostatic and π-stacking interactions (18, 20). The interactions mediated by residues Y231 and Y273 contribute to stabilizing D3, such that weakening them decreases the *T_m_* and increases the rate of pore formation, whereas replacing G274 with side-chain-containing residues greatly reduces the *T_m_*, which results in an unstable protein. Therefore, these residues of the F/Y-F/Y-X_n_-YGR motif stabilize the monomer structure of D3, whereas F230 and R275 form essential contacts with the αβ loop so that it can function to initiate the unfolding of D3 to form the pore.

We have shown that some residues of this motif can be substituted with other residues, which generally result in greater than wild-type activity. Why then are these residues so highly conserved among the CDCs and CDCLs when little else is conserved? For residues R275 and G274, the answer is relatively easy since the conservative substitutions of R275K and G274A result in much less active and/or unstable derivatives. F230A also exhibited an unstable pore-forming phenotype. The π-stacking interaction of Y231 and Y273 seems less important to the activity of PFO, since single or double alanine mutants retained ≥300% of wild-type activity. However, this increased activity comes with a loss in thermostability of 5 to 12°C, which may be advantageous or disadvantageous under specific environmental conditions (20). The glycine pair, which is also conserved, has been shown previously to be intolerant to substitution with side chain amino acids (14), which prevent the movement of the αβ loop and trap PFO in an early prepore state (32). Therefore, these residues are likely the best fit for this particular function, based on our observations. Furthermore, the central importance of this motif and that of the previously studied glycine pair (14) to the function of the αβ loop in the CDC prepore-to-pore transition explains their conservation in the CDCLs, as control of the prepore-to-pore transition is critical for CDCs to complete prepore assembly prior to the insertion of the β-barrel pore (10, 11, 18, 19, 33–36). Furthermore, the crystal structure of the *E. anophelis* CDCL exhibits a high degree of structural similarity to domains 1 to 3 of the CDCs and the residues of this motif are positionally conserved between PFO and the *E. anophelis* CDCL, and form contacts analogous to those shown herein for PFO (Fig. 7B and C), suggesting a common function.

The missing signature undecapeptide and cholesterol-binding motifs (15, 16) from the CDCLs and the lack of significant similarity between the CDC and CDCL primary structures made it difficult to know if the CDCLs were related to the CDCs. However, the studies herein clearly show the CDCLs are distant relatives of the CDCs, which likely bind different receptors. The genes encoding nearly 300 putative CDCL proteins are present in over 200 species from at least 12 different phyla that span most terrestrial niches and the microbiomes of humans, animals, and insects. This makes them the largest family of putative pore-forming toxins/proteins in the CDC/MACPF superfamily. Although most CDCL genes are present in bacterial species, genes for two putative CDCLs were identified in the genome of the diatom Thalassiosira oceanica (the genes also exhibit an intron-exon structure) and three in the genome of the fungus Basidiobolus meristosporus strain CBS 931.73. Several species also contain genes coding for more than a single putative CDCL or a CDCL plus a CDCL^S^, the extremes being exemplified by Chryseobacterium nematophagum (37) and *Chitinophaga* sp. MD30 (38), each of which have a combined total of 12 and 11 putative CDCLs and CDCL^S^s, respectively.

The *E. anophelis* CDCLs and CDCL^S^s must interact to assemble a functional pore, although on a molar basis it is 100- to 1,000-fold less active than PFO in *in vitro* assays. This is likely due to the absence of a receptor for the CDCL on the cholesterol-rich liposomes. The putative CDCL binding domains often exhibit little similarity with each other, as illustrated by the primary structures of 7 full-length CDCLs from *Chitinophaga* sp. MD30 (Fig. S7). The divergence of the binding domain 3D structure is evident in crystal structures of the *E. anophelis* CDCL and PFO (Fig. 1 and Fig. S1). The significant diversity observed among the CDCL putative binding domains suggests they may have evolved to bind targets specific to each species. Although the CDCs target eukaryotic cells because cholesterol is either the receptor or a coreceptor, it is yet unknown whether the CDCLs bind to and function on eukaryotic and/or prokaryotic cells.

Neither the CDCL nor the CDCL^S^ of *E. anophelis* exhibited any significant pore-forming activity alone, even though the CDCL^S^ readily formed CDC-like oligomers on liposomes. Mixing the CDCL and CDCL^S^ resulted in a larger SDS-resistant oligomer and exhibited higher pore-forming activity than either protein alone. Single particle analysis revealed stacked oligomeric complexes of two, and sometimes three, rings, with one exhibiting a slightly larger outer diameter than the other. This complex could be dissociated by treatment with SDS and heat, which resulted in the appearance of stable CDCL^S^ oligomers and CDCL dimers. These observations suggest the CDCL^S^ oligomer acts as a scaffold for the assembly of the CDCL oligomer. The CDCL^S^ oligomers are smaller than CDC oligomers and are composed of ∼28 monomers, versus 35 to 40 monomers for CDC oligomers (11, 13). The stacked nature of the *E. anophelis* oligomers remain an enigma, as most species encode a single CDCL gene and stacked oligomers have not been observed for other pore-forming proteins. The presence of both a CDCL and CDCL^S^ appears to be primarily associated with species of *Bacteroides*, *Elizabethkingia*, *Chitinophaga*, and *Chryseobacterium.* The stacked oligomer might interact via the top of the oligomers, thereby orienting the β-barrels of the CDCL and CDCL^S^ on opposite sides of the complex; theoretically this could insert a β-barrel into the membranes of two cells and form a channel that could facilitate the transfer of macromolecules between the cells.

These studies reveal the basis for the conservation of the F/Y-F/Y-X_n_-YGR motif and suggest that the αβ loop may be the sensor, or an important component thereof, that initiates the necessary structural interactions and transitions necessary for the prepore-to-pore transition in CDCs. The conservation of this motif and little else in the primary structures of the CDCLs and the crystal structure of the CDCL from *E. anophelis* strongly suggest that these residues and the αβ loop are similarly critical to their pore-forming mechanisms. Finally, these studies reveal that the CDCLs represent a large family of pore-forming proteins related to the CDC/MACPF superfamily and are widespread in nature.

## MATERIALS AND METHODS

### Bacterial strains, plasmids, and chemicals.

PFO and *E. anophelis* AG1 CDCL genes were codon optimized for Escherichia coli and cloned in between BamHI and NdeI sites in pet15b+ (Genscript), as previously described (19). All mutations in PFO were generated in the cysteine-less background of PFO^C459A^, referred to as wild-type PFO herein. All chemicals and enzymes were obtained from Sigma except where noted. All fluorescent probes were purchased from Molecular Probes (Invitrogen).

### Generation and purification of CDC and CDCL proteins and derivatives.

All amino acid point mutations in PFO were generated via QuikChange mutagenesis (Stratagene) prior to sequence verification at the Laboratory for Molecular Biology and Cytometry Research at the University of Oklahoma Health Science Center. The expression and purification of PFO and its derivatives and the CDCLs were performed as previously described for PFO (39). Frozen protein aliquots were thawed and centrifuged at 20,000 × *g* for 10 min and assayed for concentration prior to use in experiments.

For crystallization trials, fractions containing His_6_-CDCL were pooled and dialyzed into 20 mM Na citrate (pH 6.5), 150 mM NaCl at 21°C for 16 h. Protein was concentrated to 6 mg/ml and stored at −80°C. For X-ray crystallography, selenomethionine (SeMet) CDCL was expressed in E. coli BL21(DE3) cells using Molecular Dimensions SelenoMethionine Medium Complete, according to the manufacturer’s instructions. The SeMet CDCL was purified using the wild-type protocol.

### Affinity purification of rabbit IgG to CDCLs and Western blot analysis.

Rabbit antibody was generated (Lampire Biological Products) against purified recombinant CDCL and CDCL^S^ purified from E. coli based on the gene sequences from *E. anophelis* strain AG1. Purified CDCL or CDCL^S^ (10 mg) was coupled to 1 ml of Affigel-10 according to the manufacturer’s instructions (Bio-Rad). Unbound protein was eluted with 10 column volumes of 100 mM glycine buffer, pH 2.5. The resin was then equilibrated in HEPES buffered saline (HBS) and stored at 4°C until use. Antiserum (1 ml) was recirculated over the affinity column for 1 h at room temperature and washed with 20 column volumes of HBS. Distilled water (1 ml) was passed over the column and then the column was eluted with the glycine buffer (pH 2.5) and 1 ml fractions collected and immediately neutralized with 200 μl of 1 M Tris-HCl (pH 8.0). The antibody-containing fractions were combined and concentrated to approximately 1 mg/ml using an ultra 15-ml centrifugal filter (Amicon). Blots were probed with a 1:5,000 dilution of the antibody overnight at 4°C and then processed as previously described (10).

### Labeling of PFO derivatives with fluorescent probes.

Cysteine labeling of protein using Alexa Fluor 488 and Alex Fluor 568 or NBD was performed as previously described (14). Briefly, PFO derivatives were incubated in a 20-fold molar excess of fluorescent probe at 4°C overnight. Free dye was separated from labeled protein via G-50 column. Absorbance of the protein and label were obtained to define labeling efficiency.

### PFO modeling onto the PLY pore structure.

A model of the pore form of PFO was built on the basis of the PLY pore structure 5LY6 (13) using MODELLER (40) version 9.11. Briefly, the amino acid sequences of PFO and PLY were aligned using MUSCLE (41) and the alignment, along with the structure of a single PLY pore monomer, were used to create a homology model of PFO in the pore-monomer conformation. The PFO pore-monomer structure was read into PyMOL (42) and manually superimposed on to each monomer of the PLY pore in turn, creating a set of PFO monomers that were then assembled into a complete PFO pore.

### Liposome preparation.

Liposomes composed of 1-palmitoyl-2-oleoyl-*sn*-glycero-3-phosphocholine (POPC, Avanti Polar Lipids) and cholesterol at a molar ration of 45:55 were prepared with and without carboxyfluorescein (CF) as previously described (1).

### Carboxyfluorescein release assay.

Toxin was serially diluted (2-fold) 24 times into 96-well plates with a top concentration of 2 μM. Each toxin derivative was run in triplicate, and 100 μl of 1/1,000 CF liposome in HBS was added to each well prior to a 1-h incubation. The fluorescence emission of the individual wells of the 96-well plates was scanned using a Tecan Infinite 200Pro fluorescence plate reader (1).

### Melting temperature (*T_m_*) assay.

As previously described (19), PFO wild type and derivatives were prepared at 0.5 mg/ml with 1× SYPRO Orange (Thermo Fisher) and subjected to an increasing temperature gradient using a 7500 Fast Real Time PCR instrument (Thermo Fisher) according to the manufacturer’s protocol. Each experiment is representative of 4 replicates.

### Oligomer detection assay.

SDS-agarose gel electrophoresis (SDS-AGE) was conducted as previously described (10). Briefly, PFO or CDCLs were incubated with excess liposomes at 37°C for 30 min. Samples were then treated with SDS and boiled as indicated prior to being run on an SDS-AGE gel. The concentrations used are listed in the figure legends of each experiment.

### Negative stain imaging.

PFO mutants (0.025 mg/ml), CDCL (0.3 mg/ml), CDCL^S^ (0.05 or 0.2 mg/ml), or CDCL^S^ + CDCL (1:1 equimolar ratio, 0.05 or 0.2 mg/ml) in 20 mM HEPES (pH 7), 150 mM NaCl were applied to Teflon wells. The prereduced PFO mutants were treated with 2 mM Tris(2-carboxyethyl)phosphine hydrochloride (TCEP) reducing agent before incubation. A lipid mixture containing cholesterol:POPC (molar ratio 65:35 for PFO, molar ratio 55:45 for CDCLs) solubilized in chloroform (1 μl at 0.5 mg/ml) was then overlaid onto the wells and lipid monolayers were formed upon chloroform evaporation. Samples were incubated at 37°C for 60 min in a humidified chamber. Proteins assembled on the lipid layer were transferred onto Formvar/carbon-coated grids (mesh size 300) and stained with 1% uranyl acetate, following the method described by Rames et al. (43). All stained grids were imaged using an FEI Talos L120C transmission electron microscope (Thermo Fisher Scientific), with an acceleration voltage of 120 keV and fitted with a 4K × 4K Ceta CMOS camera at a magnification between 36,000× and 57,000× and a defocus of 0.05 to 2 μm.

### Single particle analysis.

Image processing, particle picking, and 2D classification were performed with RELION-3.0 (44). For CDCL^S^, a total of 7,678 particles from 28 micrographs (pixel size 3.1 Å) were picked and extracted with a 130-pixel box size prior to reference-free 2D classification. For CDCL^S^ + CDCL, a total of 7,018 particles from 54 micrographs (pixel size 3.8 Å) were picked and extracted with a 90-pixel box size prior to reference-free 2D classification. Dimensions of 2D class averages were measured using ImageJ (45).

### Fluorescence spectroscopic measurements.

All fluorescence measurements were performed using a Horiba Fluorolog 3 photon-counting fluorimeter in photon-counting mode with FluorEssence software. Excitation and emission wavelengths for 5(6)-carboxyfluorescein (CF) were 470 nm and 515 nm, with both slits set to 2 nm. Fluorescence scanning experiments for both NBD and Alexa Fluor 488 scanned emission from 500 nm to 600 nm with slits at 2 nm and 5 nm, respectively; excitation wavelengths were 490 nm (2-nm slit) and 480 (4-nm slit), respectively. The rate of pore formation by PFO and derivatives was observed using CF-containing liposomes as previously described (19). As toxin forms pores on the liposomes, CF diffuses out of the liposomes and its emission dequenched. The increase of fluorescence emission over time was monitored following addition of toxin to liposomes. Steady-state NBD fluorescence experiments were performed as previously described (46). Briefly, NBD-labeled toxin derivatives were incubated in the presence and absence of liposomes for 30 min prior to scanning for fluorescence intensity. Steady-state Förster resonance energy transfer (FRET) of donor dye Alexa Fluor 488 and acceptor dye Alexa Fluor 568 was performed as previously described (34). Briefly, donor-labeled PFO derivatives were incubated with liposomes in the presence and absence of a 4-molar excess of acceptor-labeled PFO derivatives. A decrease in donor fluorescence intensity signal due to FRET correlates with the formation of liposome-bound oligomeric complexes.

### Crystallization of CDCL.

Initial crystallization trials of His_6_-CDCL were set up at 21°C on a Crystal Gryphon robot (ARI, Sunnyvale CA, USA) in 96-well sitting drop format using Rigaku UV+ 96 plates (AXT, Sydney, Australia). Drops containing 0.2 μl of protein (6 mg/ml in 20 mM Na citrate [pH 6.5], 150 mM NaCl) and 0.2 μl of crystallization solution were equilibrated against 35 μl of crystallization solution. Sparse matrix crystallization screens were conducted using MCSG+ screens 1 to 4 (Microlytic, USA) with crystals initially formed in MCSG+ screen 2, condition number 42 (2.8 M Na acetate [pH 7.0]) and MCSG+ screen 2, condition number 19 (0.1 M Bis-Tris Propane:HCl [pH 7.0], 2.8 M Na acetate [pH 7.0]) at 21°C. Both crystallization conditions were scaled up using the hanging drop vapor diffusion method in Linbro culture plates (ICN Biomedical, Inc., Aurora, OH, USA) at 21°C. The crystals were cubic with the largest dimensions of up to 120 μm^3^. No cryoprotection was required before freezing in liquid nitrogen.

### Data collection, structure determination, and refinement.

CDCL crystals were diffracted to 2.09 Å, without cryoprotectant, on the MX2 beamline of the Australian Synchrotron (Clayton, Victoria). Data collection was controlled using Blue-Ice software (47). For phasing, diffraction data were acquired from a single CDCL crystal containing SeMet residues in place of normal methionine. Two data sets were acquired, one at the measured selenium peak for the crystal of 12867 eV and the other at 13000 eV. Each data set consisted of a full 360° rotation being 720 images with a crystal oscillation of 0.5°. Data were processed with XDS (48). The crystals belong to space group *P*4_2_2_1_2 with unit cell parameters of *a* = *b* = 121.74 Å, *c* = 88.91 Å. Experimental phases were determined using the peak wavelength data set alone with hkl2map (49) in SAD mode. Autotracing in SHELXE found 361 residues of the 514 residues with unambiguous positioning of six of expected eight selenomethionine residues from the anomalous selenium signal. An additional data set for a native CDCL crystal containing methionine was acquired to 2.2 Å resolution, without cryoprotectant, on the MX2 beamline of the Australian Synchrotron and merged with the selenomethionine data set for final refinement. The structure was refined by iterative rounds of manual rebuilding in COOT (50) and refinement with Phenix (51) and BUSTER (52). Final refinement statistics are summarized in Table S2.

10.1128/mBio.02351-20.2 TABLE S2 X-ray diffraction data collection and refinement statistics. Download Table S2, DOCX file, 0.01 MB. Copyright © 2020 Evans et al. 2020 Evans et al. This content is distributed under the terms of the Creative Commons Attribution 4.0 International license.

### Accession numbers.

Atomic coordinates and structure factors of have been deposited in the Protein Data Bank under the ID code 6XD4.
