# Supplementary figures and images for: A Key Motif in the Cholesterol-Dependent Cytolysins Reveals a Large Family of Related Proteins
Source: mBio. 2020 Sep 29;11(5):e02351-20. doi: 10.1128/mBio.02351-20 (PMC7527733; doi:10.1128/mBio.02351-20)

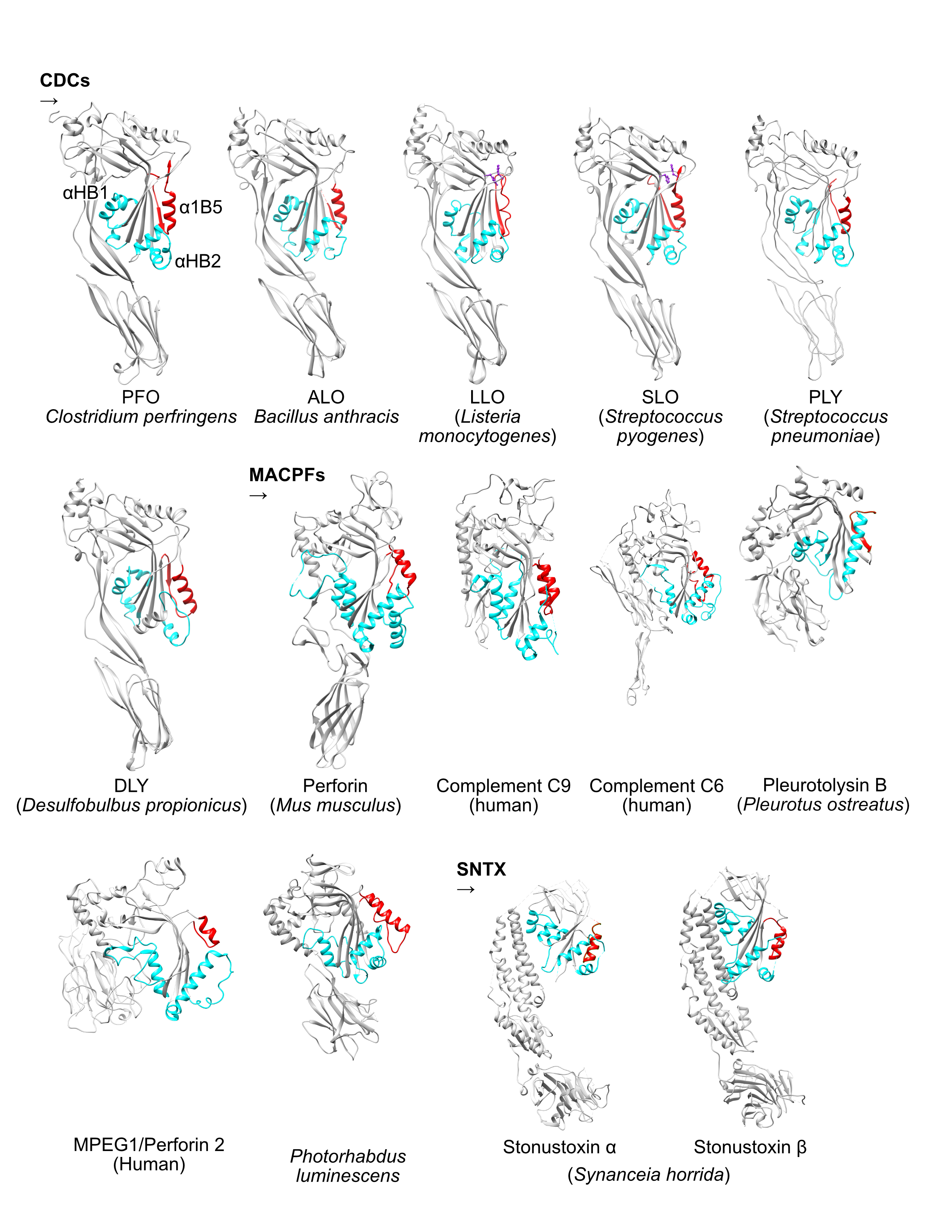

Supplement: FIG S1 [file mBio.02351-20-sf001.tif]

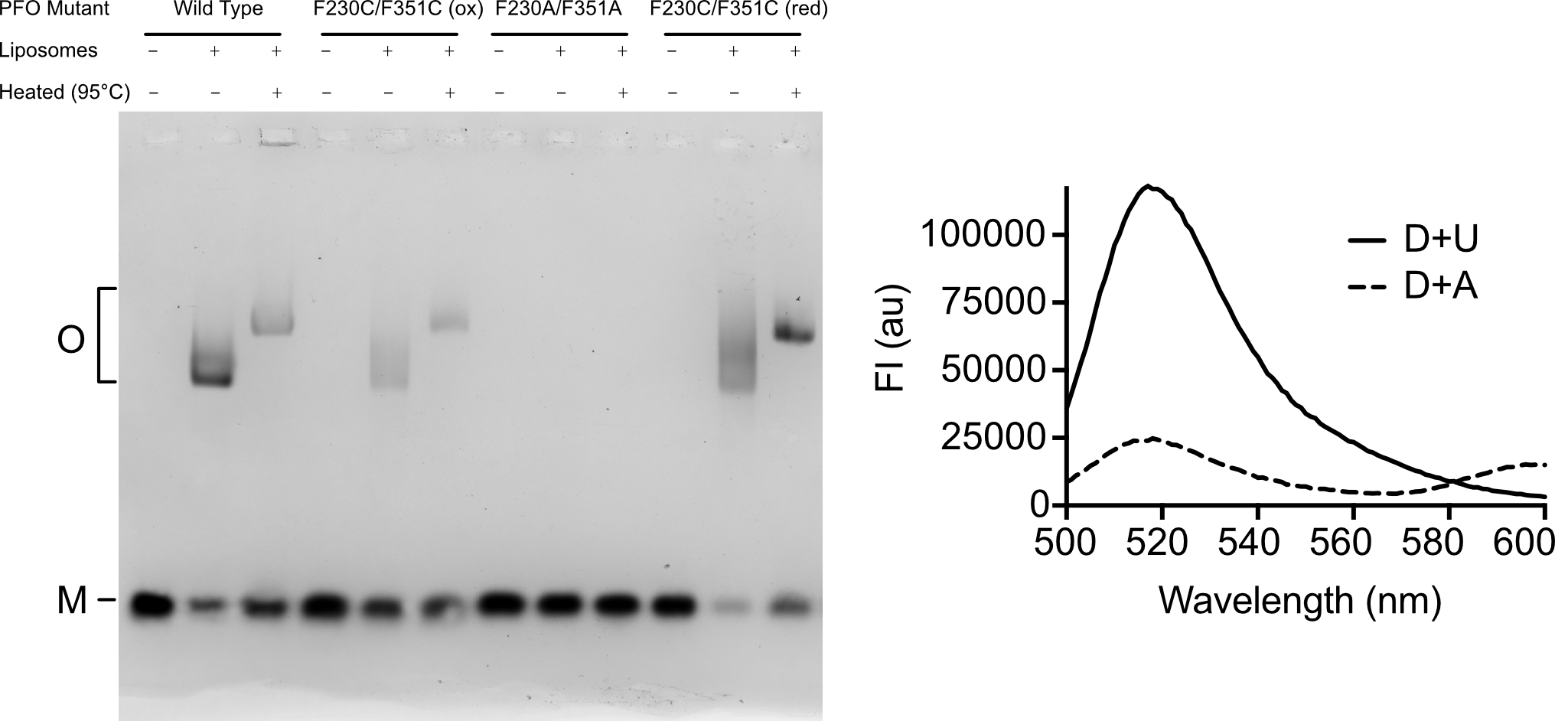

Supplement: FIG S3 [file mBio.02351-20-sf003.tif]

Figure S4.


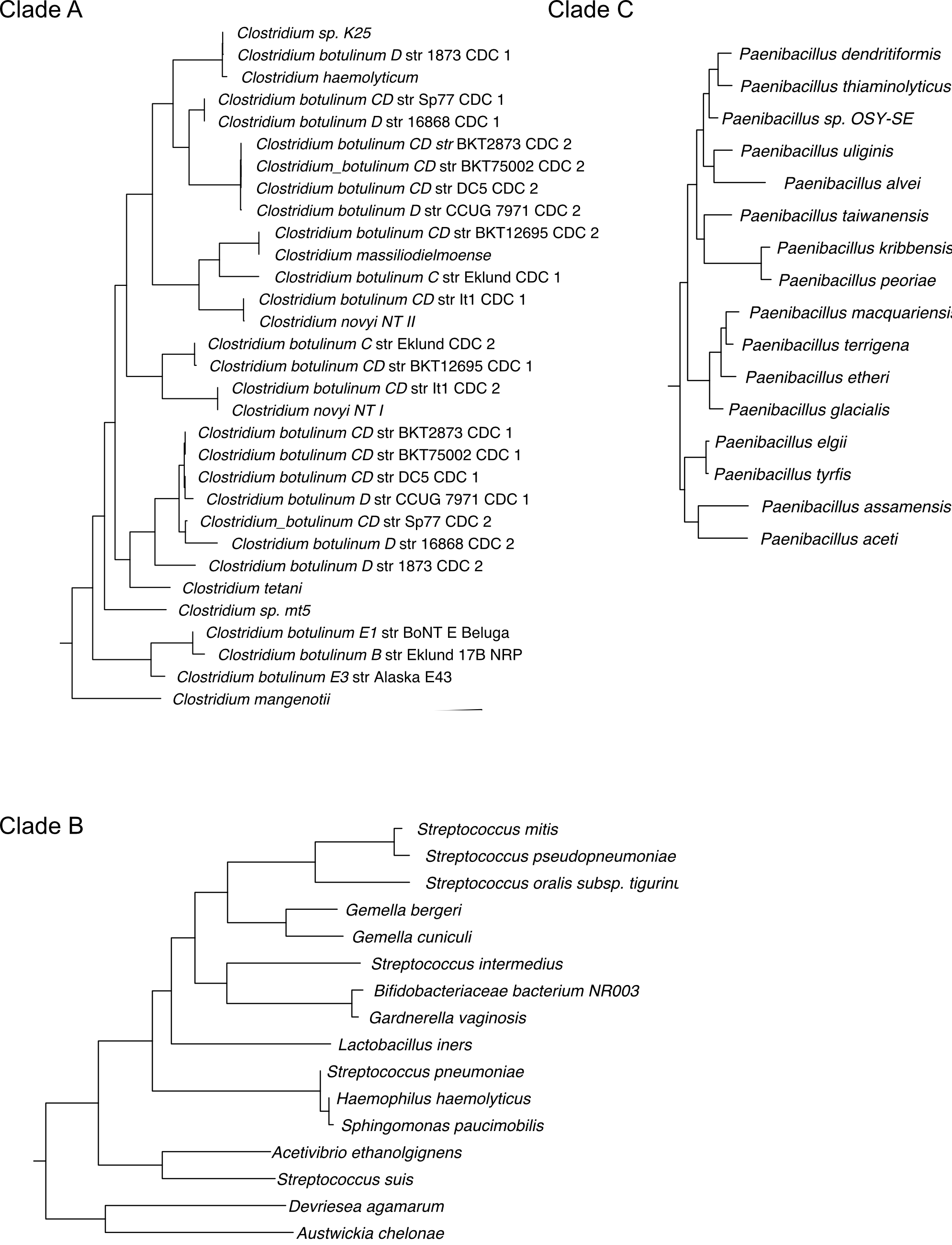


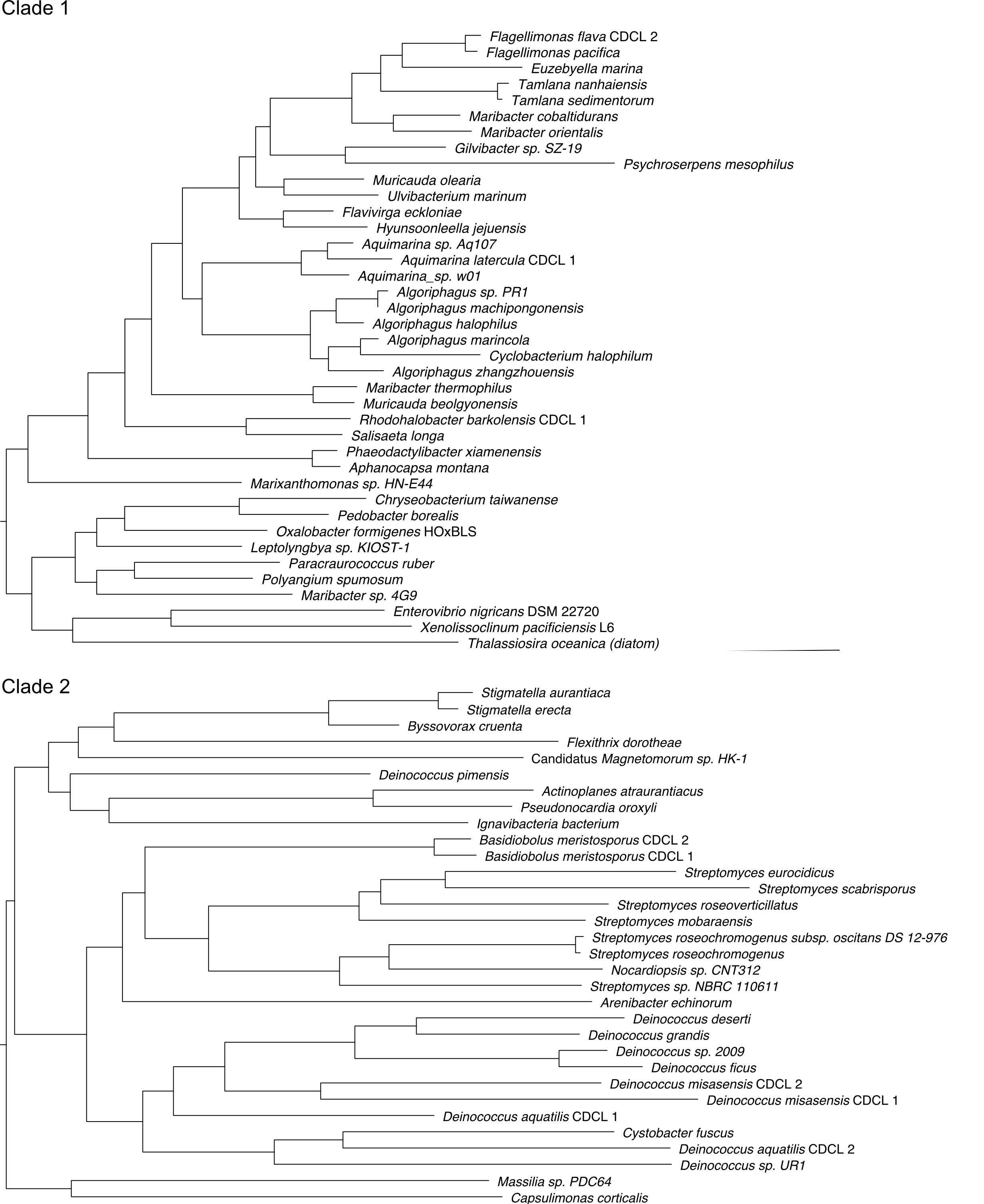


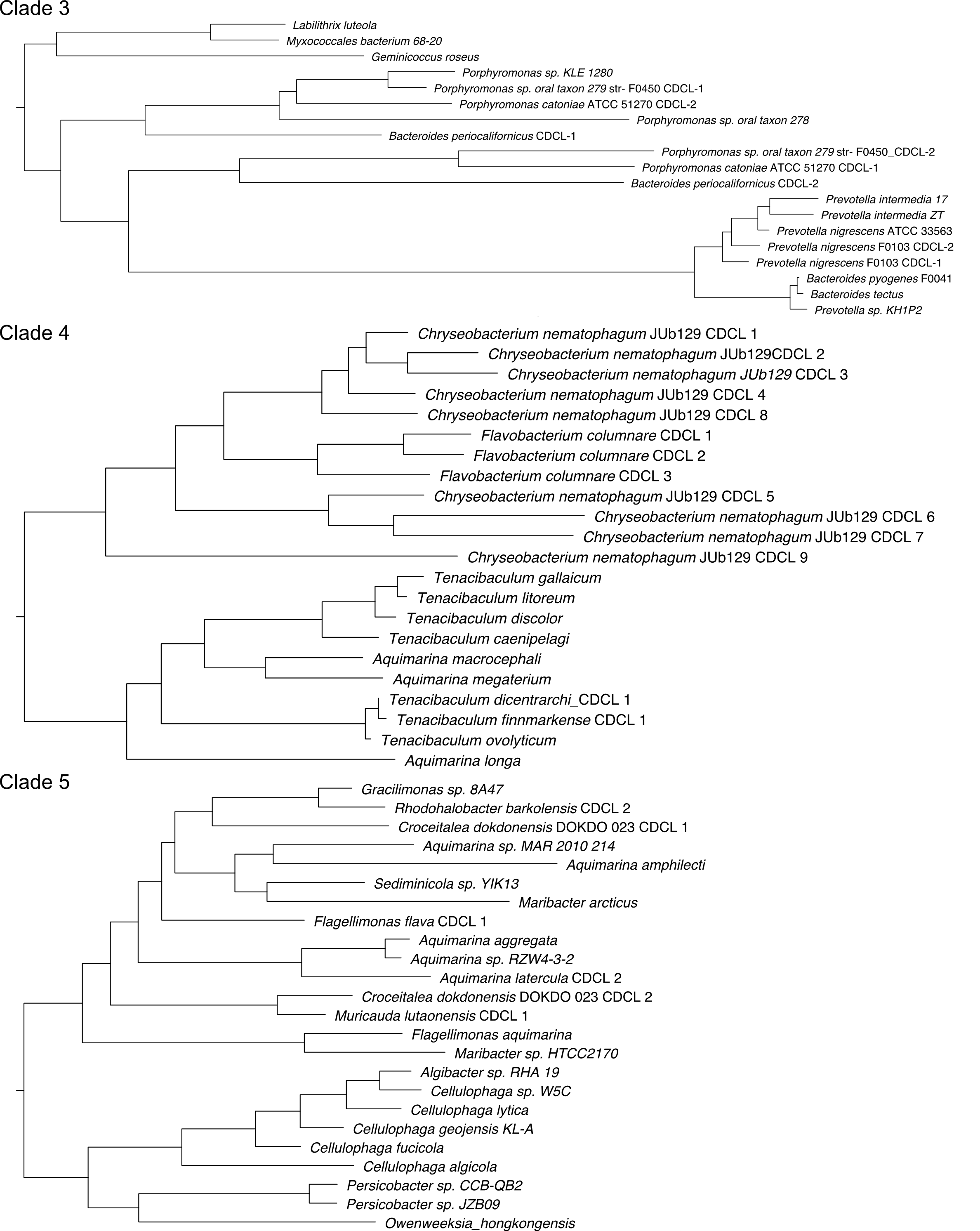


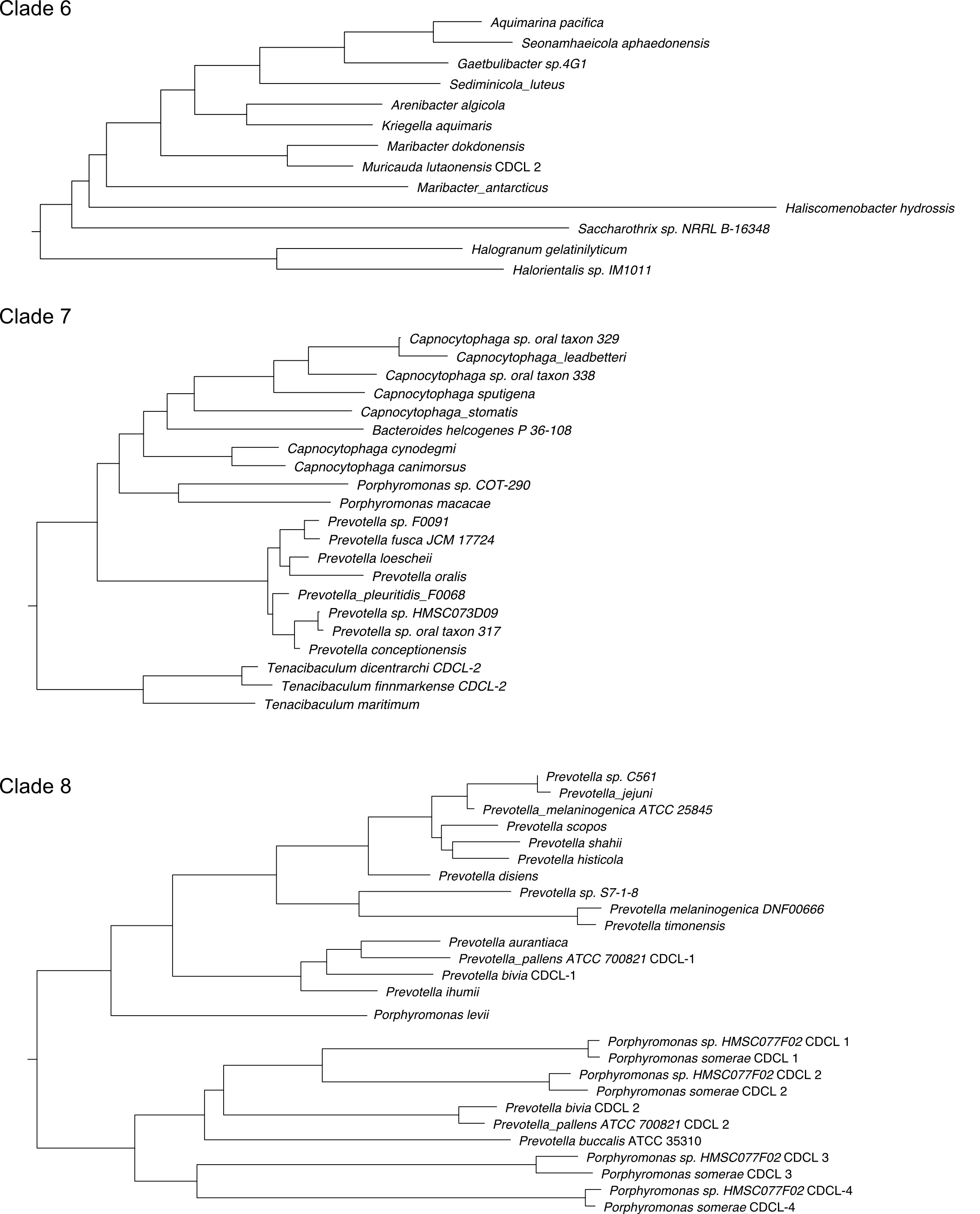


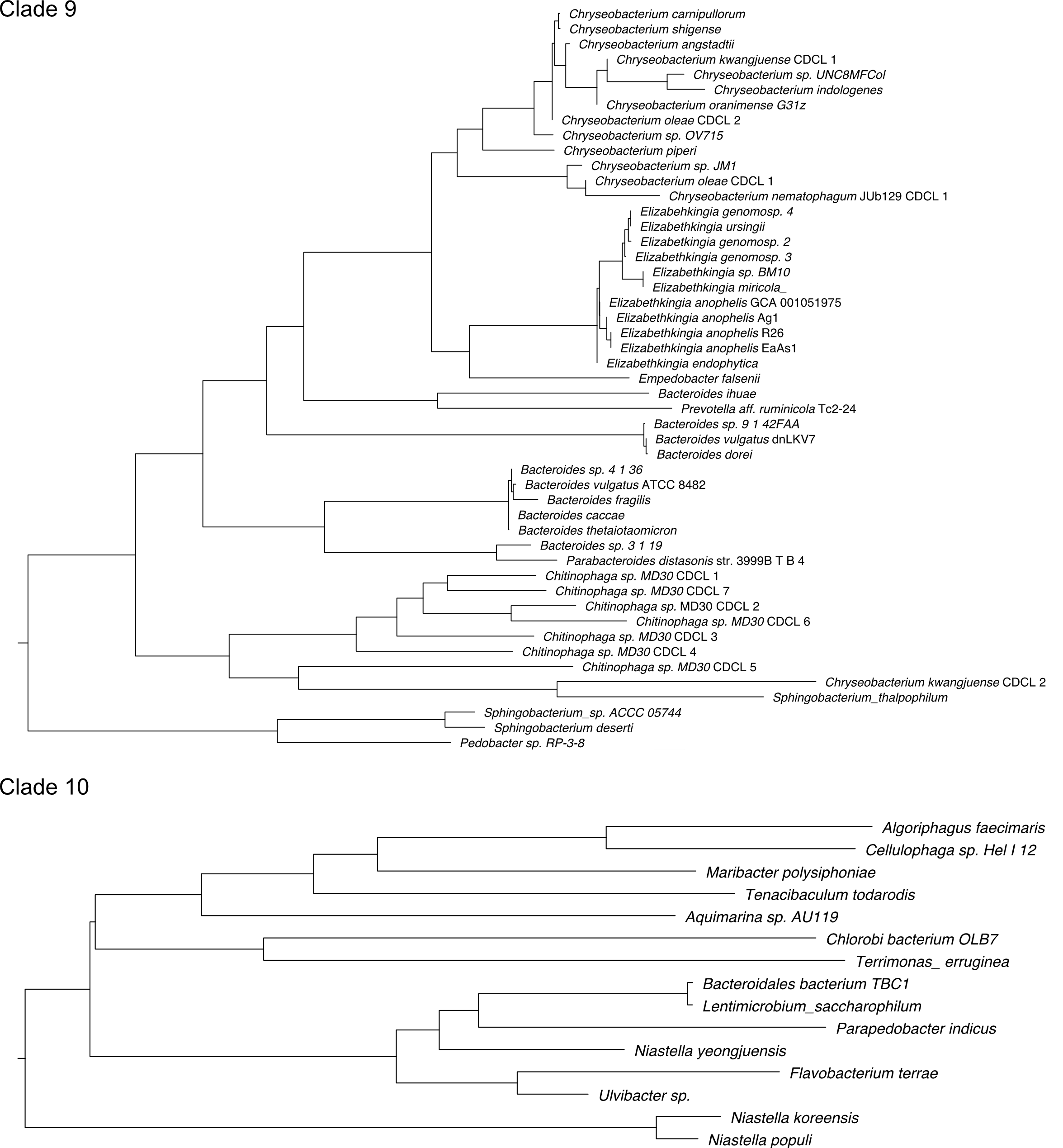

Supplement: FIG S4 [file mBio.02351-20-sf004.docx]

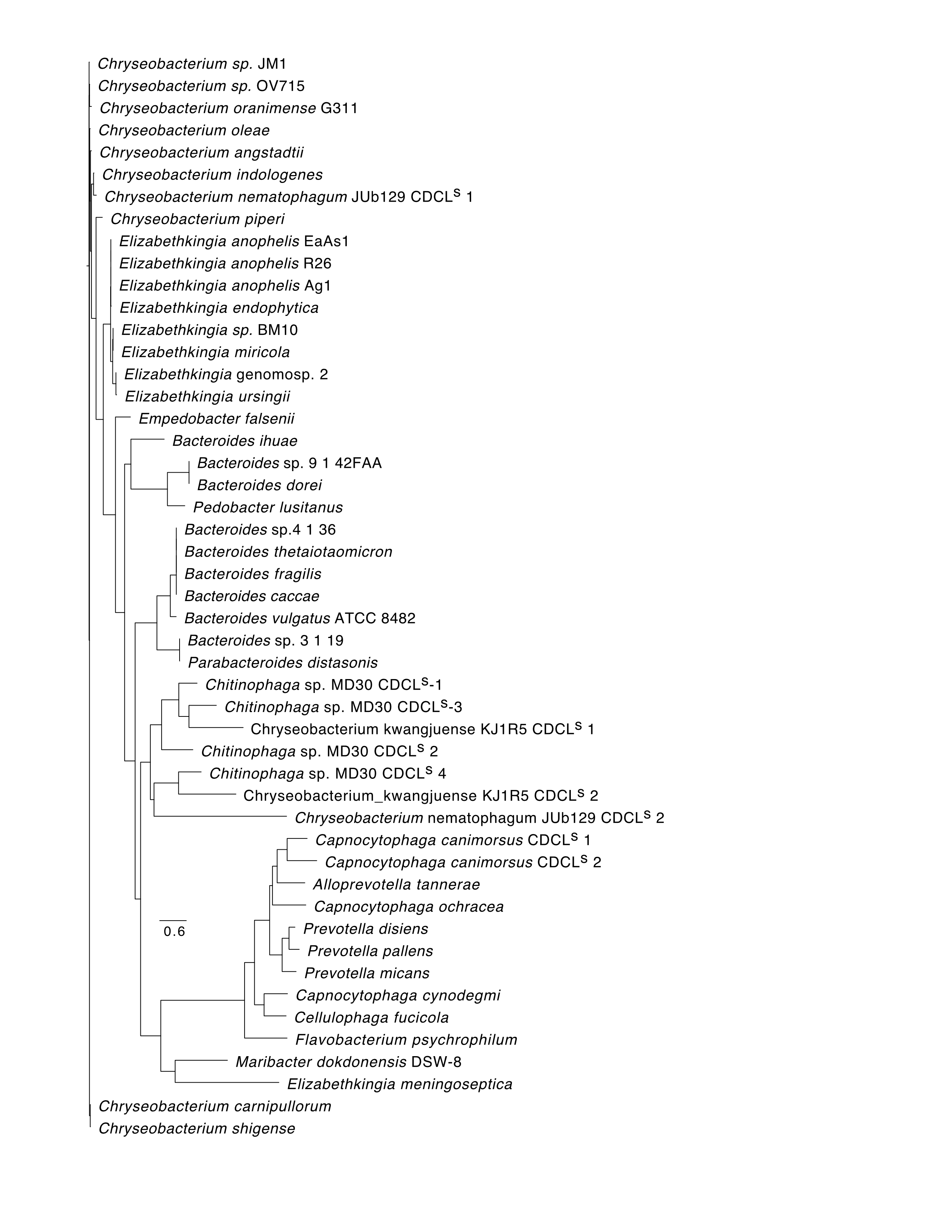

Supplement: FIG S5 [file mBio.02351-20-sf005.tif]

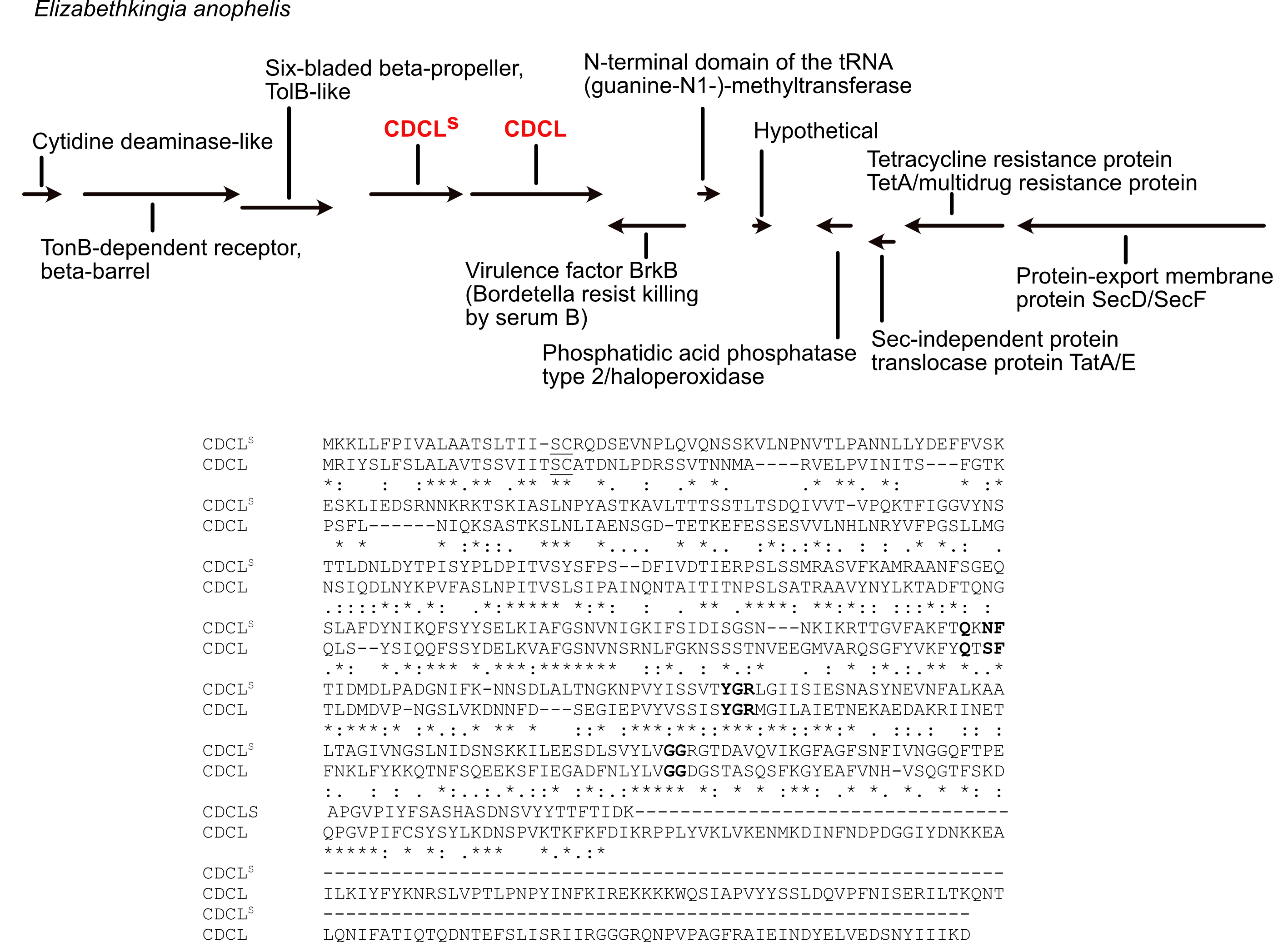

Supplement: FIG S6 [file mBio.02351-20-sf006.tif]

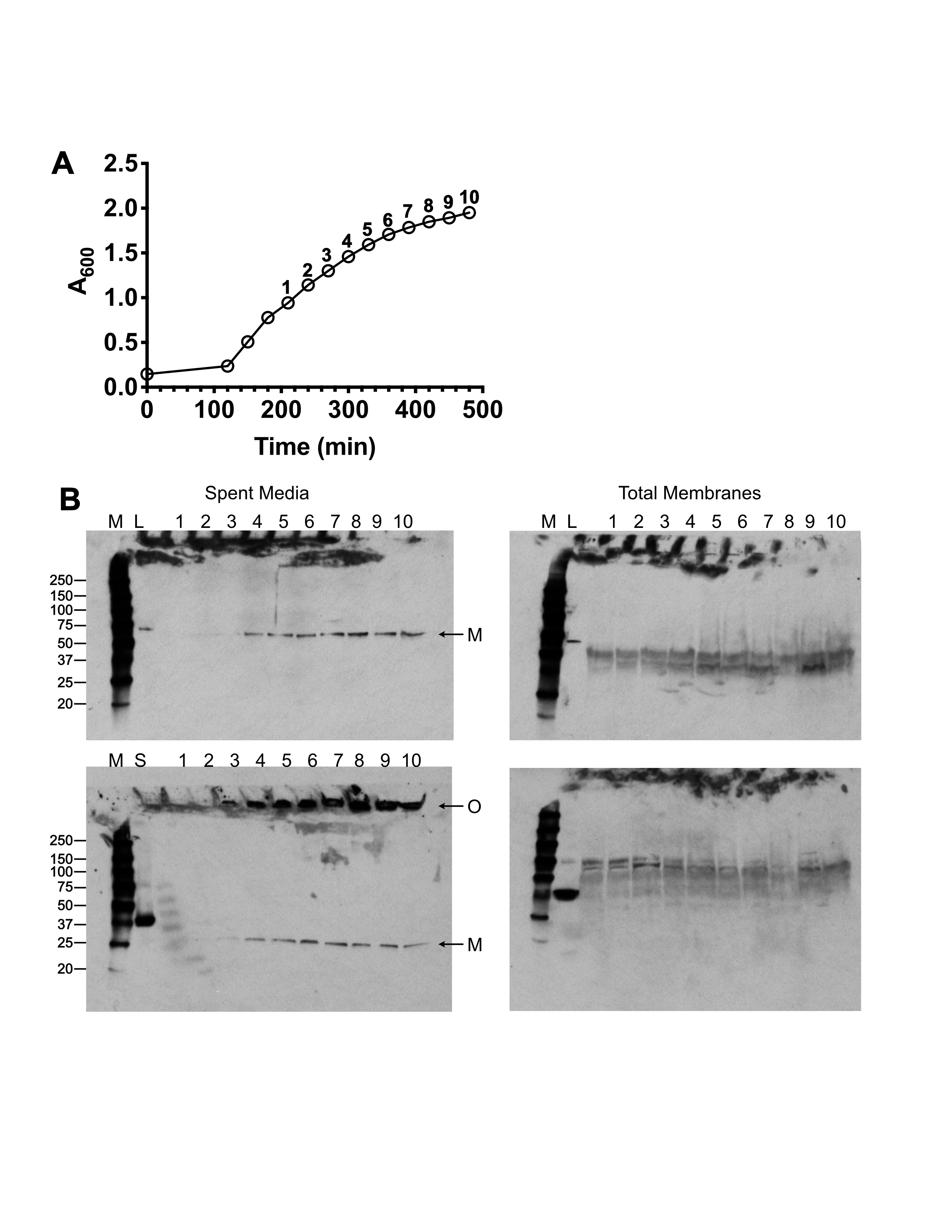

Supplement: FIG S8 [file mBio.02351-20-sf008.tif]
