# Supplementary material for: A Key Motif in the Cholesterol-Dependent Cytolysins Reveals a Large Family of Related Proteins
Source: mBio. 2020 Sep 29;11(5):e02351-20. doi: 10.1128/mBio.02351-20 (PMC7527733; doi:10.1128/mBio.02351-20)
Supplement: FIG S2 [file mBio.02351-20-sf002.docx]

Figure S2

*^1^Rhodohalobacter barkolensis*  MNRLK----------NKTTLQTKIKEFI--------------------------------

*^2^Salisaeta longa**  MI------------------------FF--------------------------------

*^3^Aphanocapsa montana*  MTF----------------------RFT--------------------------------

*^4^Labilithrix luteola*  M------------------------KLV--------------------------------

*^5^Thalassiosira oceanica**  MMSSW----------SREPPGPDPTR----------------------------------

***Clostridium perfringens***  MIRFK----------K--------TKLI--------------------------------

*^6^Maribacter arcticus*  MNSTKKNMKTLQNLRNKFTSNWGMRSVL--------------------------------

*^6^Prevotella melaninogenica* ATCC25845 MKKYLF------------------KAVL--------------------------------

*^7^Ignavibacteria bacterium*  MKKY--------------------------------------------------------

*^8^Halogranum gelatinilyticum* MSDESSETSVRRRTLLATVSGLLSASLAGCSVSIDTPFSGSTGSGQSTPQPTPVTRTRTR

*^9^Capsulimonas corticalis**  MTDLTDLEKIRQLVL-------------------------------SIPIPPP-------

*^7^Bacteroides vulgatus* ATCC8482 MR----------------------------------------------------------

*^7^Elizabethkingia anophelis* Ag1 MR----------------------------------------------------------

*^10^Deinococcus deserti**  MVLASFLHSWGGAQV---------------------------------------------

*^5^Basidiobolus meristosporus**  MFFEALVNA---------------------------------------------------

*^11^Streptomyces mobaraensis**  MAQAASLEKVELPDL---------------------------------------------

*^12^Treponema vincentii* ATCC35580 MQKQKRAKLTARIKRYIVLLICLAAAGIAGCQQPAKPLVKEIKTEKPIQKPATPSSQKPD

*

*Rhodohalobacter barkolensis*  ------------------------FLPACLLLVLLSSCSDNSV-----SGE--E----PQ

*Salisaeta longa*  ------------------------IATGC------DSGSGMTE-----PGQ--R----PD

*Aphanocapsa montana*  ------------------------ALLCLLSLFLFQSCNKDEEEMPAPPKE--N----PM

*Labilithrix luteola*  ------------------------ALSMLAAVVACGGASPEGDLTAAPEAA--NAPSAPS

*Thalassiosira oceanica*  -----------------------------------PPTAPDSSAISDASAQ--LASRWP-

***Clostridium perfringens*** ------------------------ASIAMALCLFSQPVISFSKDITDKNQS---------

*Maribacter arcticus*  ------------------------FFGAFLVLLLIASCGKDDPIATDPIADTPEEPIALT

*Prevotella melaninogenica* ATCC25845 ------------------------PVSMAVCAITFGACSQDELLTPVKEQSEEGVLNNP-

*Ignavibacteria bacterium*  -------------------------LAILISILIFYSCSDTTGPT--------------K

*Halogranum gelatinilyticum*  EPTATATATPTQAPTTEEPTTATPAASTSTASPTTTTTTTPNSQVADETPSFDPPPCLAD

*Capsulimonas corticalis*  --------------------------------ETFESTSPDNLPVGVKATSYQNGP---D

*Bacteroides vulgatus* ATCC8482 ----------------ILRYTLFLFAIG------LISCEQE-------------------

*Elizabethkingia anophelis* Ag1 ----------------I--YSLFSLALAVTSSVIITSCAT--------------------

*Deinococcus deserti**  ------------------------------------RPLP-----VQPIQPMRPAPVIIQ

*Basidiobolus meristosporus*  ------------------------------------------------------APV---

*Streptomyces mobaraensis*  ------------------------------------PALREHQYVIAAFTGDGSLPVAVA

*Treponema vincentii* ATCC35580 KQVKPDKSDKPDTGKG--------------------------------------------

*Rhodohalobacter barkolensis*  GVDEVGDFLANLTYDAD------------ELLNV---------Q--PNDAA-R-------

*Salisaeta longa*  GAS-IGEYLSGLTYDAD------------ALLNV---------Q--PSDAA-R-------

*Aphanocapsa montana*  ATD-INAYIRSLAYDPV------------EMLNV---------NNTPGDLAQK-------

*Labilithrix luteola*  APSDNAAPSNEGAADQA------------NAINALVASAGHMSTDKQADAEPV-------

*Thalassiosira oceanica*  -APGIKEYIESLDYNAD------------EILEV---------------RTEE-------

***Clostridium perfringens*** ----IDSGISSLSYNRN------------EVL-----------------ASNG-------

*Maribacter arcticus*  ISEVVNGGNKNLLQKKS------------ELI---------FNEELIGTSVDK-------

*Prevotella melaninogenica* ATCC25845 --KEIRNYLKTLPLAP---------------MMTRANGPINPDDGAPIPVEEE-------

*Ignavibacteria bacterium*  PAKDIDDLVRNAGVVNT------------SVV------------------TEK-------

*Halogranum gelatinilyticum*  GTCGGPPTNRNITFDPSVVSQLNRSLIDPSVLNVDLTTSSETSSDPPSATTNSGAVSVEV

*Capsulimonas corticalis*  G-------------------------LD-EVVNIGSKIASQLAT-----ITYLGA-----

*Bacteroides vulgatus* ATCC8482 --------FDKKEQQSDCCSFQNLPKYPEQVVNI-----------------NVHNYIPDM

*Elizabethkingia anophelis* Ag1 ---------DNLPDRSSVTN--NMARVELPVINI-----------------TSFGTKPSF

*Deinococcus deserti*  PLRPLPTTLRQLDQLPRAELMNMITLANIQFTQIP---------------RPTLIIPLLL

*Basidiobolus meristosporus*  ---DLPSDRSQINDY-FATLPDWDKFANVQSSD---------------------------

*Streptomyces mobaraensis*  ---PVGVGDRQAKADVAAALAKWRKWRELSPYQPN---------------AK--------

*Treponema vincentii* ATCC35580 -----ENTNGTQNGNNSGNGSNEQEGISYKGLNIPDSLAR----------SPQKLRELIV

↓P104-G105

*Rhodohalobacter barkolensis*  --EP-VDTT-ATTEQDGITQRTCTSTTYTLQNNFEE--VAILRPTQDVIW**PG**ALVEANQS

*Salisaeta longa*  --TP-IDTT-TTTEQDGTVERTCVRTTYNLQTNFEE--IAILRPTADVIW**PG**ALVEANQS

*Aphanocapsa montana*  --TPGASDT-ETGPLDRGYRTTCVRQSYELDRDFSE--IAILNPGSGNIY**PG**ALVLANAD

*Labilithrix luteola*  --QKGEPTT-TQVPGEGGTTYSCTYTDYDLTKVPEK--FVALNPNADVLW**PG**SLVQG-KS

*Thalassiosira oceanica*  --DKKQEEI-SYDEEDGKTFKT-TRVEYTVTKNLKK--LPILGNLEGVIF**PG**NLVRADSK

***Clostridium perfringens*** --DKIESFVPKEGKKAGNKFIVVERQKRSLTTSPVD--ISIIDSVNDRTY**PG**ALQLADKA

*Maribacter arcticus*  --SDDVTTASEDGADIITQRYICTNQRVSLLDGTAD--FNLLNSNPEVIY**PG**NLLQG-KT

*Prevotella melaninogenica* ATCC25845 --NPIVETQGVLDGIPGNWVKTTRH--YKIKQTFDE--NFLFDPTSDVVY**PG**CVLKGG-T

*Ignavibacteria bacterium*  --DEKVSEETQLNVLEGVKKWNIKLTKKSLAWNLDNNIIPFNNPNANTLW**AG**ALVQGNEV

*Halogranum gelatinilyticum*  TPDQKQTETTSQGESSTTENDGMVCTVRKKTATTGGSDVFLLNPSMTEVY**PG**ALLTAASI

*Capsulimonas corticalis*  ------------------------------------------NPDV--IW**PG**ALVQCQYL

*Bacteroides vulgatus* ATCC8482 GDKVLSSPSHIHTRASEIISETLWDNGFGKTIHAQADESIVLDNLNRYIY**PG**SILDGASV

*Elizabethkingia anophelis* Ag1 ----LNIQKSASTKSLNLIAE---NSGDTETKEFESSESVVLNHLNRYVF**PG**SLLMGNSI

*Deinococcus deserti*  PPITRVGPATQVVQDTPDGQVACSVQRYSLRAAPPE--HAMKTLDQDKLW**VG**SLVRTA-G

*Basidiobolus meristosporus*  ---TIKGDVKSQEASMGSVQLECSVQDITLTDTPKE--LVTFGTGSDAFW**LG**ALLQGK-G

*Streptomyces mobaraensis*  ---SKVGP----EEVTHVKGVKRTKQTYSLTKNPEE--AITFDPNVSMCF**PG**AIVQAKPA

*Treponema vincentii* ATCC35580 QGAQLSETEPQFNNHGAVVKYIEKEVEYDISAAFDD--TLLLDPSQNSIY**PG**AVLRGDSL

: * :

*Rhodohalobacter barkolensis*  LLDGLPEPARF-ERS---------PVKIRIDLP-GI---GTNGTKTIESPDQ----ANVQ

*Salisaeta longa*  LLDGLPEPARF-DRA---------PVKIRVDLP-GI---GENGTKTIEEPDL----ASVQ

*Aphanocapsa montana*  LLSGTPRPLGL-ERN---------PVTLTIDLP-GI---GEEGSFTVPEPSN----ATVS

*Labilithrix luteola*  MAGGILDPIPV-KRA---------PGTITLTLASGG---GGPFFKRMESPSL----SEAM

*Thalassiosira oceanica*  LVDGKPSALTHQDRT---------KCTLTITLP-------KEETSSQVYPSF----SHVH

***Clostridium perfringens*** FVENRPTIL-MVKRK---------PININIDLPGLK---GENSIK-VDDPTY----GKVS

*Maribacter arcticus*  LNQAPPLPI-VVKRG---------GGTISYDLVNA----NTTSFYNVEEVKK----SSIT

*Prevotella melaninogenica* ATCC25845 IANGTYAMITSHKTG---------DVTFSISLSPAN---PREAHET--SATV----PNIR

*Ignavibacteria bacterium*  PNGILNSIGDNLSRT---------PLTITVQ--SGS---GNLGNNIIEKPSN----ANYS

*Halogranum gelatinilyticum*  ADGSFAPALTSQRRGGVSTQTIRNPLQLSISLANID---GTN-TKTVQTPSL----GAVR

*Capsulimonas corticalis*  SKST--PDLLNIERA---------PGRIALSMYQLD---GSK-SPNYEAPVVDMTPGAVR

*Bacteroides vulgatus* ATCC8482 ANQDYKTISVHYK-----------PINVSVSFP-A-----QKVTGVLEKPSLSSCRQLVM

*Elizabethkingia anophelis* Ag1 QDLNYKPVFASLN-----------PITVSLSIP-AI---NQNTAITITNPSLSATRAAVY

*Deinococcus deserti*  LELGSMTAINI-------PEDRRNPYRVT-SALSTV---SGSATI---APNQTAYNLAAA

*Basidiobolus meristosporus*  YVDGVGGFKPL-------PIGDRNEQKITVDFLNGK---SSSSIC---KPDSGAVRDAIA

*Streptomyces mobaraensis*  IENGYLIPAGI-------EDSDRADLGITVDRLTSR---KETAS----PPSASNVTAAIG

*Treponema vincentii* ATCC35580 DRESYQEITEGNKRNAV----------ISFDLQGVKDKEGKDGKAGVTSGEIIPDLASYR

.

*Rhodohalobacter barkolensis*  NAIDEALEWWNANAYEEGYVNAASSSNRITTSYASTQASLDLGLNVEWAT--GDV--QSQ

*Salisaeta longa*  TAIDEALEWWNANAYEEGYVNAASSSNRITTSYSSTQASLDVGLNVAWAT--GDV--QSQ

*Aphanocapsa montana*  PAIDEALEHWNNTAFQQGYFNGARSYYEASEAFEQRQLGIELGINAEWVT--GSV--ASH

*Labilithrix luteola*  QAQNEILASYTG-------ATPAKFSYSFQSIYSSEQLAVAVDANVRGTN--WSA--SAA

*Thalassiosira oceanica*  EFIQANEKAWFES--HGGSVEAGSLDISCVEAHSKEQVSFSLGLKGQMGN--DSI--STL

***Clostridium perfringens*** GAIDELVSKWNEK-YSSTHTLPARTQYSESMVYSKSQISSALNVNAKVLE--NSL--GVD

*Maribacter arcticus*  DGMNQIIAQAVDLEF------PDNLTMEVIDIDSESHLAIEIGISVETFA--TKT--KGN

*Prevotella melaninogenica* ATCC25845 KS--EYQEVWNKWATMDWKESPVTTIQSVEKINSQEELVTKLGVAVTAPV--ANG--SVN

*Ignavibacteria bacterium*  KVLQDILDKNVTKC-------TAKQYLTVQIAHSKQQACIKLGFSANWLT--GSL--STN

*Halogranum gelatinilyticum*  TARNEILDRVGSGA------TPAVMSYEKQRIYSKDQ--LDVELGAHFSN--PSVDVSGS

*Capsulimonas corticalis*  TALSTILAQKAAPE------QPARLDKQYTTFYSLEQSTLSMGLSAHWLS--DSM--KAS

*Bacteroides vulgatus* ATCC8482 DLMHQK----------GIGQQSASVHFDIHRFTSYDELKMTFGSNANTSFLFWGSSSSQQ

*Elizabethkingia anophelis* Ag1 NYLKTA----------DF-TQNGQLSYSIQQFSSYDELKVAFGSNVNSRNLFGKNSSSTN

*Deinococcus deserti*  AVRQGMVGNPF----------GSTVRYEITEQSSAETSALKLGLKAGGI----GYSVKAA

*Basidiobolus meristosporus*  GLVSHAKSGGYR--------PGANIHYKTVEVNSAESAALNLGFGAKYL----AFQAKTA

*Streptomyces mobaraensis*  KVVGDDAPG------------SSDVVFRRVEAYDSSETALELGISAKYG----GFAASLD

*Treponema vincentii* ATCC35580 ELRNKILSQNIT------YHASAHSSYEEMEITNEKSLEAQLKMGVGFGAAGIKTKIAAG

.

Q228↓ ↓F230-Y231

*Rhodohalobacter barkolensis*  FNFETSEESRVVMATFK**Q**A**FY**TITYVQENGAQPEDVFGPSVSLQQV-QSA----FSSTAP

*Salisaeta longa*  FNYETTETKRVVMATYK**Q**A**FY**TVSFVQESGAQPEDVFGPEVTLQAV-QAA----FDSDAP

*Aphanocapsa montana*  LSTSTSTDRRVALLAFR**Q**I**FY**TIT--AQPPFEPTDYLGPDVTLEQV-QSS----TDANNH

*Labilithrix luteola*  LSVDKNDEKSRFLIEFS**Q**E**YF**TMAFDPPQGA--AGVFDPSVTAKDL-EPY----AAAGNP

*Thalassiosira oceanica*  LNVTQESEKRVCFAKVQ**Q**V**FY**SVVVDCPKS--PETVFADSLSIEDA-KSA----FSSESP

***Clostridium perfringens*** FNAVANNEKKVMILAYK**Q**I**FY**TVSADLPKN--PSDLFDDSVTFNDLKQKG----VSNEAP

*Maribacter arcticus*  FSFSTDKTYNRKLVKLQ**Q**V**FY**TMTYDFPNSY--EEVFDDSVTAEQLAK-Y----IQPDNP

*Prevotella melaninogenica* ATCC25845 LGFNFNKKKNHILARLI**Q**K**HF**TVSTDAPKK---------GTIFESIDKDA----LDGYQ-

*Ignavibacteria bacterium*  FETNNSSEQKSIYLLFK**Q**V**YF**TVSVNEPTK--PSDYFGDNIKLEDL-NYF----VKPNNP

*Halogranum gelatinilyticum*  FDFTQTTETNKLLATFS**Q**K**YY**DISVSLPNPAGNGVV--SDMSY--LRKND----VIVNN-

*Capsulimonas corticalis*  LDLSTSTKKTRILVHFR**Q**S**YY**EVSFE-KDPGITSVF--ANTAKFEIAKLE----MGIDNP

*Bacteroides vulgatus* ATCC8482 EHKERISKSSGLYIRFI**Q**K**YF**TIDMDIPEK---SFI-EGSINAQ-Y-------------S

*Elizabethkingia anophelis* Ag1 VEEGMVARQSGFYVKFY**Q**T**SF**TLDMDVPNG---SLVKDNNFDSEGI-------------E

*Deinococcus deserti*  GSLSSENRQNRVSAAFV**Q**N**AF**TMNADLGGRS-ATEAFLKNPTAEDLAAVT-----SPGSP

*Basidiobolus meristosporus*  LDTQRNYTENTIAAYFV**E**K**AF**TVDVAMP-KT-PADFFNSQFTMQDLKEQIRFGRLGPDNL

*Streptomyces mobaraensis*  ISARRKETMNTVLVYLR**E**R**GF**TAFCDVS--T-PGALFKDSFTEEKLNKLVSGGYMGPDNP

*Treponema vincentii* ATCC35580 FKFKNGEQKERRLIKFV**E**T**FY**TVDVNQEAAPMMINI---------------PREVVGDRM

: :

↓Y273-R275

*Rhodohalobacter barkolensis*  PAYVSSIT**YGR**IIMFRMETSSSYTSAEVETAF---RYAAGG-QVEGDLESTYKEILQNSS

*Salisaeta longa*  PAYIASVT**YGR**IIMFRMETASSYTAAEVEAAF---KYAAGT-QVDGDLEARYQEILSSST

*Aphanocapsa montana*  PAYVSAVD**YGR**IILFRMEVTNVETSIDLNAVM---NYAAGGVSVAADVASRYDEILSNSS

*Labilithrix luteola*  PVYVSGVT**YGR**IFYILFESSASQTSLEAAVKG---SYSGPSIGADASASASWKKLINEST

*Thalassiosira oceanica*  PAYIGQVA**YGR**LTVIKMETKGSYSAVDMEVSM---QSMIEGVKVDGTFKSTYESIKENSS

***Clostridium perfringens*** PLMVSNVA**YGR**TIYVKLETTSS--SKDVQAAF---KALIKNTDIKNS--QQYKDIYENSS

*Maribacter arcticus*  ATFISSVS**YGR**IFYMLIESTSSKTEMDLKLKG---EYNGALTNVEAEGHINSFNQLKETK

*Prevotella melaninogenica* ATCC25845 PVYISSIN**YGR**IIYLSIETDEKERNINEAIEFALNKIKGVDVNVSVDQAVNYRKMLAKSD

*Ignavibacteria bacterium*  LCYVASVD**YGR**LILVKMTYNENITSTELDAKV---KLAFSAYEVGGDYKTT--DIESKST

*Halogranum gelatinilyticum*  ------VS**YGR**LLFFSVESKYTYQEVETALDV---AVKSGLNKVEADLSTADKQVLKNTK

*Capsulimonas corticalis*  PGYVSYVL**YGR**ELVLAITSDAEENDVRAALDV---SYSGIVAGGDAHISATQQSILNSLS

*Bacteroides vulgatus* ATCC8482 PVYVSSIA**YGR**VGILTLETDEIYENAE---SIVKKAVNGFLYNKKEFLTVEEKGFFDEAG

*Elizabethkingia anophelis* Ag1 PVYVSSIS**YGR**MGILAIETNEKAEDAK---RIINETFNKLFYKKQTNFSQEEKSFIEGAD

*Deinococcus deserti*  AAYIDSIT**YGR**LLFVEMTSSYTSQQMKAALDA---SYSG----VSASAQAESQKVLSDSR

*Basidiobolus meristosporus*  PIFVSSIS**YGR**VLLYSVTSSASSSDIRATIQA---SYNGLKLGVDSNLDIKHQKLLSESK

*Streptomyces mobaraensis*  PLLVNSVV**YGR**IIVFTFTSTSSETEIKAALEASYHGFA-DVDAHLKAHYLGIISKSEVSI

*Treponema vincentii* ATCC35580 PVYVSSVS**YGR**IAYLTIESDQEKSELKANLDTVFKVTAAN--NVEADIDTAIKKLEKGTT

: *** .

↓G324-G325

*Rhodohalobacter barkolensis*  VEVITI**GG**NAAVASEAVTARSA-G---DLVPIITGDNAVYSRNNPGV**PI**AYAVKYLKDDK

*Salisaeta longa*  VEVVTL**GG**NAAVASEAVTARSA-G---DLVPIITGENAVYSRSNPGV**PI**AYAVKYLKDDQ

*Aphanocapsa montana*  ISVLTI**GG**SAQSAAQALDNISGPG---SLTPLIV-ENAVYSRENPGL**PI**AYRVNLLSDNR

*Labilithrix luteola*  VKAYGL**GG**NAEMAINAVTGASQFD---KIATFLT-SGANFSTQNPGV**PI**SYTIRHLTDSS

*Thalassiosira oceanica*  FSLIAF**GG**NAKETIEVFKDFTQFK---AYLT----EGANLTESNRPK**PI**SYTTFFLKDNS

***Clostridium perfringens*** FTAVVL**GG**DAQEHNKVVT--KDFD---EIRKVI-KDNATFSTKNPAY**PI**SYTSVFLKDNS

*Maribacter arcticus*  VKIIAY**GG**DGSEGMSGLRTTNE----------ITKRLEETKNITLGV**PL**SYTVRSLEDPA

*Prevotella melaninogenica* ATCC25845 VHITVL**GG**GKTIQQEILKG--DID---SFQRFLAADIPMEQM----Y**PI**SFSLRYAVDNS

*Ignavibacteria bacterium*  FECLIL**GG**SNGGAAKALGSMN-LN---GILDLIKAE-SEYTPNTPAY**PI**SYVVKNLADNS

*Halogranum gelatinilyticum*  VNINVL**GG**SASSASKLISGYGDTGAAAAIGDWIQRGATYSPSDSPGA**PV**AYQTKYLSGLQ

*Capsulimonas corticalis*  ITVGVI**GG**SGQAAADLVAIQNPT--MADVATFIRAGATVGPN-SPAE**PL**TYKVNWLLNDQ

*Bacteroides vulgatus* ATCC8482 MKVYVG**GG**NGDSGVKTLTGFDDFIKFIS-------EGGHFSAETPGK**PI**FCSFAYLSDHS

*Elizabethkingia anophelis* Ag1 FNLYLV**GG**DGSTASQSFKGYEAFVNHVS-------QGT-FSKDQPGV**PI**FCSYSYLKDNS

*Deinococcus deserti*  FNVYAA**GG**SEQAVVDLIRTQK-----------LAQYFRDTSDPRTLV**PI**SFTARNFTDG-

*Basidiobolus meristosporus*  ISVAVI**GG**NREAAINLIRDGN-----------LNSFFSQESTLETYV**PI**SYTLRSIANG-

*Streptomyces mobaraensis*  ISKGST**GG**QIKELLTKGT-----------LAESFATPQKYKSYVRIG**YT**LQTL-DG-IPA

*Treponema vincentii* ATCC35580 ITINII**GG**GSEAVTDLKQFQK----------YIVKEG--FSSKNPGH**II**KYQLRFLDDNA

. ** . : .

PFO D4→

*Rhodohalobacter barkolensis*  V-AKLGYTTEYTATEC----------------------***SAI----RTADVVTVDLIEFEA***

*Salisaeta longa*  L-AKLGYTTEYTATEC----------------------***SSV----QTINTITVHLKEFYV***

*Aphanocapsa montana*  I-VKLGFSSDYTDVRC----------------------***GSI--------AYNHPRIDFY-***

*Labilithrix luteola*  Q-VRLALTTEYTAKNC----------------------***TPM-------------------***

*Thalassiosira oceanica*  L-ATIGDACKWVESVR----------------------***KEVFVGWRNRGAFTA-RFSFLY***

***Clostridium perfringens*** V-AAVHNKTDYIETTS----------------------***TEYSKG----------------***

*Maribacter arcticus*  ILVGVKLATEYDKVTCVLKGELAPTNYRGLVDVFEDGI***GAAFQLEGTTIVVYNKAGDRYA***

*Prevotella melaninogenica* ATCC25845 Q-ARVVTSNEFTVTQRDFVPVFKKVRMQLQVLGFSGQH***SGPLP-----------------***

*Ignavibacteria bacterium*  I-VKLGESTEYTVKEYTENREDYQTFDFYLSQFNIIDD***GNFW------------------***

*Halogranum gelatinilyticum*  T-ANVYLSTTYNARTCRPKTRRFRVHNFDIRVVTANDV***VGSKNTEELYGSIF--------***

*Capsulimonas corticalis*  T-AAFNFVDNYNITT---RSSAPLIKDISITFVTSADK***----------------------***

*Bacteroides vulgatus* ATCC8482 -PYKVKFKIDIDSDPVYARIEYRNLK------------***----------------------***

*Elizabethkingia anophelis* Ag1 -PVKTKFKFDIKRPPLYVKLVKENMKDIN---------***----------------------***

*Deinococcus deserti*  TYAASSTTGEFAESVCNP-NSVKAWVR--VSYRSIE--***----------------------***

*Basidiobolus meristosporus*  NLAAVSETTKYTIKACHPVRRGYKWTFKIVSLKLID--***----------------------***

*Streptomyces mobaraensis*  KMSETTNYDAVV---------WGDSTVTLKVKS--***-------------------------***

*Treponema vincentii* ATCC35580 T-AYIKYGEKYKTVERVEIPAKGYKVTATVENIKGAGF***EAAHITGTVGMQPLDRDTPRKT***

*Rhodohalobacter barkolensis*  ***VNDCD--VGPGDFDFNATVYYNDEQMFNTGGV--G-----PWI-SAELDDGETKSID---***

*Salisaeta longa*  ***RKDCDGIEGDGEFEFRAIV--------NGGGTRAG-----DFIREATLGDGGRVRLN---***

*Aphanocapsa montana*  ***----------NDFD--------------------------------------TYNLR---***

*Labilithrix luteola*  ***TKGCDGVLGSGKTTDKCGVCGGDGTTCD----PCG-----PTTLQHRAGNGAYVNFNVGS***

*Thalassiosira oceanica*  ***KKKSDGPDGPDH-------------KVKTDWIPCG-----GRKGKFSLGNAYDIRFKVDV***

***Clostridium perfringens*** ***------------------------------------------------------KINLDH***

*Maribacter arcticus*  ***LYTVGGGNAPFKYYVSDPFGPIASIVSTTGTIGAAVRWRDGRIHLFNTEGNSFLKFRYDP***

*Prevotella melaninogenica* ATCC25845 ***----------------------------------------------NLDKDANIWGKVLV***

*Ignavibacteria bacterium*  ***--------GDGKFFYKIQIIDKN-----------GNLLKDVNDKDIIIQTDKFNYIKVGD***

*Halogranum gelatinilyticum*  ***------------------------------------------------------------***

*Capsulimonas corticalis*  ***------------------------------------------------------------***

*Bacteroides vulgatus* ATCC8482 ***------------------------------------------------------------***

*Elizabethkingia anophelis* Ag1 ***------------------------------------------------------------***

*Deinococcus deserti*  ***----------------------PEDSKYDDV---FGEVALDGTGIWSLGSGNRVDLYKGQ***

*Basidiobolus meristosporus*  ***----------------------PAEPASDDTAEAWGDIVLNGQYIWSVEKPRYQSIRAGQ***

*Streptomyces mobaraensis*  ***-------------------LGTPF--GTRESF-DITIDGQELPGA---------SGHPAT***

*Treponema vincentii* ATCC35580 ***IFNYSGTETPSKLTIKRGTGAKPDAQSDSIIVP-------------------------DE***

*Rhodohalobacter barkolensis*  ***----EE---VVFNAQRQD--GNTFAVDFWAREQDNPPFG----------S----PNYNDL***

*Salisaeta longa*  ***----EE---VVFDIERQD--GNEFGITFYSTEWDKKIWG----------EVFKDPNMATV***

*Aphanocapsa montana*  ***----IK---LTYD-------------------HDYPPAD----------D----------***

*Labilithrix luteola*  ***GNHGDR---VTFP----D--GNYY--KYAKALCNRVIWG----------NVSLVCHDGSW***

*Thalassiosira oceanica*  ***MFAVDQWKCIIDDKLPSP--NNKYLTAFGTTIVPQWEWE----------N----------***

***Clostridium perfringens*** ***SGAYVAQFEVAWDEVSYDKEGNEVLTH---KTWDGNYQD----------KTAHYSTVIPL***

*Maribacter arcticus*  ***ENSNLTTPTGSFGDIELDSDGKPKVFFTNLYYGDATIFVGSFPFDANGVEAAVQYTHSSN***

*Prevotella melaninogenica* ATCC25845 ***GVNGQEHELVGIDK------SNPFWFDYREKEETLHPIGFGGIVNVEFDKDKNESLEDFV***

*Ignavibacteria bacterium*  ***GGKITLTNGIIKGVALKKQSGEQF-------TVSAQIWDYDADTDLEIHQAGKQGNIYNY***

*Halogranum gelatinilyticum*  ***---------VGGRAYSKDGSNSQPIIGLDSWGADSNNWVRV-KQGQSKDLNIDNTLEFST***

*Capsulimonas corticalis*  ***---------------DKDDDSYLRISITRSDGAEVASWRQTEKDGFEKGST--RTITFSP***

*Bacteroides vulgatus* ATCC8482 ***-----NDSYVGRSLQREKIIGDVHLAFYADRTAKIPTVAPRYISFNIVEHSRHYLKA---***

*Elizabethkingia anophelis* Ag1 ***----FNDPDGGIYDNKKEAILKIY--FYKNRSL-VPTLPNPYINFKIREKKKKWQSIAPV***

*Deinococcus deserti*  ***TLNLYRAAQP---------------LT-LNY-------G---------------------***

*Basidiobolus meristosporus*  ***DVNFIKSPKATVFTDLADDVSPPFSLY-LNFIDDDTWNG---------------------***

*Streptomyces mobaraensis*  ***AYRAFAE-------DGSGDPYRITCMKYPVIGTTDT---------------------***

*Treponema vincentii* ATCC35580 ***SCAIQLSFAIKGEMVILGKINGVQQVFVRYPQGDRP------------------------***

*Rhodohalobacter barkolensis*  ***NGKKKRTH------TYNINTGWTNLDPGNGAPDKDGDGVQDLEIELVGE--------KSN***

*Salisaeta longa*  ***RSVKR--H------TFG-STGWSNV-PTSG------------DIRLVNG--------SQN***

*Aphanocapsa montana*  ***-------------------TGETT------------------ELQINDE--------SSA***

*Labilithrix luteola*  ***TWSGQQSFDSDHLCTSDKNNGWSS-----------------------GD--------DSI***

*Thalassiosira oceanica*  ***------------------------------------------------------------***

***Clostridium perfringens*** ***EANARNIRIKARECTGLAWEWWRDVISEYDVPLTNNINVSIWGTTLYPG--------SSI***

*Maribacter arcticus*  ***ELSDQRYFSNGGTDTATLRDRIENADYLPQWLWGNPESIVDYELTILPNVGAATTVVSGA***

*Prevotella melaninogenica* ATCC25845 ***DHQKISFITDLHTENGIYKYNYGRTQF---------------------NHTLGTVFSKYK***

*Ignavibacteria bacterium*  ***-------------------PSWNND---------------------FNNWIYMNLVPDAG***

*Halogranum gelatinilyticum*  ***EGELDRRKSYIEV----VMEPREHDAA--GDEFK-----GARKSVRWFLSESPSDPDRA*G**

*Capsulimonas corticalis*  ***F-----RIFYVHDLTIGVCNAMYHLSTNGGDEWQFTTEIAATTSSGLTFIMLPEVADWM*G**

*Bacteroides vulgatus* ATCC8482 ***------RIPRT------EKDDTTVEEFVKSNNSRGTELQLKHDLLLTEFVWTHNNPRRT*R**

*Elizabethkingia anophelis* Ag1 ***YYSSLDQVPFNISERILTKQNTLQNIFATIQTQDNTEFSLISRIIRGG---GRQNPVPA*G**

*Deinococcus deserti*  ***--------------------QAR-TMTLTGRLMDWDAGSPNDVIGVWNEAIDLRAVAEE*L**

*Basidiobolus meristosporus*  ***--------------------GFD-STIVNGNFTEWEVGT---------HTMKLRSEGEM*L**

*Streptomyces mobaraensis*  ***-----------------EAHVDWRLSPKELGWFAGGTTTASGRYPET------GNV*W*MDY***

*Treponema vincentii* ATCC35580 ***------------------------------------------------QPSIRTVSTFK*N**

*Rhodohalobacter barkolensis*  ***TCRVKVYYDVT-----------------LQ***

*Salisaeta longa*  ***-CQAELVYSVG-----------------VM***

*Aphanocapsa montana*  ***TLP-------D-----------------VP***

*Labilithrix luteola*  ***SCGWQ------------------------P***

*Thalassiosira oceanica*  ***------------------------------***

***Clostridium perfringens*** ***TYN---------------------------***

*Maribacter arcticus*  ***NTRSDIYFSVAGDQMAIKKAGAWSGPYFIN***

*Prevotella melaninogenica* ATCC25845 ***SDNPVVVLECNYKHIKIHTYVKILDLKFFN***

*Ignavibacteria bacterium*  ***DYNIQFIYRIEKK-----------------***

*Halogranum gelatinilyticum*  ***SGRGRFKKRFADRGTEIEITFDITPLPPR-***

*Capsulimonas corticalis*  ***D-----KK--TDSGPH--------PLIYQG***

*Bacteroides vulgatus* ATCC8482 ***YEQNTFR-YSLSEGGFYKILPAINEPKKWR***

*Elizabethkingia anophelis* Ag1 ***FRAIEINDYELVEDSNYIIIKD--------***

*Deinococcus deserti*  ***KSTDLVRREFRKRGEADADGVMIVEFSRNN***

*Basidiobolus meristosporus*  ***LTYTITKEKFG---------------TPEK***

*Streptomyces mobaraensis*  ***EAVKDG-------------------NS***

*Treponema vincentii* ATCC35580 ***SSLQQFMLHREGYPNETLVFDVRFKVEKEP***
