# Supplementary material for: A Key Motif in the Cholesterol-Dependent Cytolysins Reveals a Large Family of Related Proteins
Source: mBio. 2020 Sep 29;11(5):e02351-20. doi: 10.1128/mBio.02351-20 (PMC7527733; doi:10.1128/mBio.02351-20)
Supplement: TABLE S1 [file mBio.02351-20-st001.docx]

**Table S1.**

| Residue | Interactive  residues | IE (kJ/mol) | Residue | Interactive  residues | IE kJ/mol  (∆WT) |
| --- | --- | --- | --- | --- | --- |
| **PFO** |  |  | **F230A** |  |  |
| F230 | F351 | -16.2 | F230A | F351 | -4.4 (-11.8) |
|  | R275 | -9.0 |  | R275 | -0.2 (-8.8) |
|  | G274 | -9.0 |  | G274 | -1.2 (-7.8) |
|  | Y273 | -6.8 |  | Y273 | -2.4 (-4.4) |
|  | I229 | -6.2 |  | I229 | -2.9 (-3.3) |
|  | N228 | -5.2 |  | N228 | -0.4 (-4.8) |
|  | A357 | -4.1 |  | A357 | -0.6 (-3.5) |
| F351 | F230 | -18.5 | F351 | **A**230 | -11.1 (-7.4) |
|  | T353 | -10.6 |  | T353 | -10.5 (-0.1) |
|  | L175 | -9.5 |  | L175 | -9.3 (-0.2) |
|  | S352 | -8.8 |  | S352 | -8.8 (0) |
|  | Y231 | -6.9 |  | Y231 | -7.0 (+0.1) |
|  | T350 | -3.3 |  | T350 | -3.3 (0} |
| **F351A** |  |  | **F230L:F351L** |  |  |
| F230 | F351**A** | -7.9 (-8.3) | F230L | F351**L** | -8.0 (-8.2) |
|  | R275 | -8.9 (-0.1) |  | R275 | -2.8 (-6.2) |
|  | G274 | -8.8 (-0.2) |  | G274 | -1.7 (-7.3) |
|  | Y273 | -6.4 (-0.4) |  | Y273 | -6.4 (-0.4) |
|  | I229 | -6.2 (0) |  | I229 | -6.6 (-0.4) |
|  | N228 | -5.0 (-0.2) |  | N228 | -3.8 (-1.4) |
|  | A357 | -0.9 (-3.2) |  | A357 | -2.8 (-1.3) |
| F351A | F230 | -4.3 (-14.2) | F351L | F230**L** | -10.1 (-8.4) |
|  | T353 | -0.6 (-10.0) |  | T353 | -7.7 (-2.9) |
|  | L175 | -3.0 (-6.5) |  | L175 | -8.4 (-1.1) |
|  | S352 | -1.3 (-7.5) |  | S352 | -6.2 (-2.6) |
|  | Y231 | -0.3 (-6.6) |  | Y231 | -2.8 (-4.1) |
|  | T350 | -3.0 (-0.3) |  | T350 | -3.9 (+0.6) |
| **F230A:F351A** |  |  | **PFO** |  |  |
| F230A | F351 | -1.8 (-14.4) | Y275 | N348 | -16.2 |
|  | R275 | -0.2 (-8.8) |  | G325 | -9.8 |
|  | G274 | -1.2 (-7.8) |  | D326 | -6.5 |
|  | Y273 | -2.4 (-4.4) |  | N228 | -5.8 |
|  | I229 | -2.8 (-3.4) |  | V322 | -5.3 |
|  | N228 | -0.4 (-4.8) |  | G324 | -3.2 |
|  | A357 | -0.6 (-3.5) | **Y275A** |  |  |
| F351A | F230 | -2.8 (-15.7) | Y275A | N348 | -2.7 (7.1) |
|  | T353 | -0.6 (-10.0) |  | G325 | -2.1 (1.1) |
|  | L175 | -3.0 (-6.5) |  | D326 | -1.3 (4.0) |
|  | S352 | -1.3 (-7.5) |  | N228 | -1.3 (4.5) |
|  | Y231 | -0.3 (-6.6) |  | V322 | -0.5 (15.7) |
|  | T350 | -3.0 (-0.3) |  | G324 | -0.4 (6.1) |

| Residue | Interactive  residues | IE (kJ/mol) | Residue | Interactive  residues | IE kJ/mol  (∆WT) |
| --- | --- | --- | --- | --- | --- |
| **PFO** |  |  | **Y231A** |  |  |
| Y231 | Y273 | -12.9 | Y231A | Y273 | -0.8 (-12.1) |
|  | F230 | -9.7 |  | F230 | -2.5 (-7.2) |
|  | I133 | -9.4 |  | I133 | -3.1 (-6.3) |
|  | L135 | -8.3 |  | L135 | -3.5 (-4.8) |
|  | F351 | -6.2 |  | F351 | -0.6 (-5.6 |
|  | V162 | -5.2 |  | V162 | -0.2 (-5.0) |
|  | I158 | -4.6 |  | I158 | -0.1 (-4.5) |
| Y273 | Y231 | -14.6 | Y273 | Y231A | 0.0 (-14.6) |
|  | V271 | -12.5 |  | V271 | -12.6 (+0.1) |
|  | P359 | -10.8 |  | P359 | -10.9 (+0.1) |
|  | A272 | -8.8 |  | A272 | -8.8 (0) |
|  | V233 | -8.0 |  | V233 | -7.9 (-0.1) |
|  | Y358 | -7.9 |  | Y358 | -7.9 (0) |
|  | A357 | -5.9 |  | A357 | -6.0 (+0.1) |
| **Y273A** |  |  | **Y231A:Y273A** |  |  |
| Y231 | Y273 | -5.9 | Y231A | Y273 | -7.0 (-5.9) |
|  | F230 | -9.7 |  | F230 | +1.9 (-11.6) |
|  | I133 | -9.4 |  | I133 | -1.1 (-8.3) |
|  | L135 | -8.3 |  | L135 | -1.9 (-6.4) |
|  | F351 | -6.2 |  | F351 | -1.0 (-5.2) |
|  | V162 | -5.2 |  | V162 | -0.1 (-5.1) |
|  | I158 | -4.6 |  | I158 | -0.1 (-4.4) |
| Y273A | Y231 | -5.0 | Y273A | Y231 | -5.9 (-8.7) |
|  | V271 | -0.7 |  | V271 | -3.0 (-9.5) |
|  | P359 | -1.4 |  | P359 | -2.7 (-8,1) |
|  | A272 | -2.9 |  | A272 | -1.0 (-7.8) |
|  | V233 | -0.3 |  | V233 | -0.4 (-7.6) |
|  | Y358 | -2.5 |  | Y358 | -5.3 (-3.6) |
|  | A357 | -2.4 |  | A357 | -1.1 (-4.8) |
| **PFO** |  |  |  |  |  |
| G274* | F230 | -10.4 |  |  |  |
|  | I360 | -6.5 |  |  |  |
|  | Y358 | -6.0 |  |  |  |
|  | G325 | -4.4 |  |  |  |
|  | N228 | -4.0 |  |  |  |
|  | G324 | -2.8 |  |  |  |
|  | R275 | -2.7 |  |  |  |
|  | Y273 | 0 |  |  |  |
| G274A | F230 | -4.6 (-0.5) |  |  |  |
|  | I360 | -2.1 (+0.2) |  |  |  |
|  | Y358 | -5.9 (-0.1) |  |  |  |
|  | G325 | -4.1 (-0.3) |  |  |  |
|  | N228 | -2.0 (-2.0) |  |  |  |
|  | G324 | -0.1 (-2.7) |  |  |  |
|  | R275 | +23.6 (-26.3) |  |  |  |
|  | Y273 | +7.2 (-7.2) |  |  |  |
