# Supplementary material for: A Key Motif in the Cholesterol-Dependent Cytolysins Reveals a Large Family of Related Proteins
Source: mBio. 2020 Sep 29;11(5):e02351-20. doi: 10.1128/mBio.02351-20 (PMC7527733; doi:10.1128/mBio.02351-20)
Supplement: FIG S7 [file mBio.02351-20-sf007.docx]

*Chitinophaga* sp. *MD30* CDCL-1 MRKYYSLSAILLLMLLLGNACRKASKEEQTAPISSSALTA-FSHLKKYPERTIQIDLKGE

*Chitinophaga* sp. *MD30* CDCL-7 MKTIIS-PVVLVLLCCLSSSCHKQELEKTVSPVAGDVLSA-FQHMKKYPEQPIQINVKDR

*Chitinophaga* sp. *MD30* CDCL-3 MSRIPFL--WLLLSTAILYSCKKEA-----EPMSAAKESAGFSHLKKYPEQLIQIDLRDQ

*Chitinophaga* sp. *MD30* CDCL-2 MSTHYKL-VLGVAAILLATSCQKKEKEAM-QATLSGQALNAFRHLPKYPQQTITIDLKDQ

*Chitinophaga* sp. *MD30* CDCL-6 MKSIYSA-VGLLVLCCLSNSCRKQEMDKLIDKSAAEQALD--------------------

*Chitinophaga* sp. *MD30* CDCL-4 MK-IYSMILLTGMSCLLLAACQKKEQELTQLPVSGNVLSA-FSKMPKYPEQMIRIQVKN-

*Chitinophaga* sp. *MD30* CDCL-5 MFAIKRLFIFAVVLALL-SACRKPKADV----IGEQQDRYSFSQLPRYHLTPFKVKGK--

* : :*:*

*Chitinophaga* sp. *MD30* CDCL-1 APRSPFNGV-AVDAGKVLDEQKLS-DPV--PG--TIGYVNPIRELI---NWETGSYAWEF

*Chitinophaga* sp. *MD30* CDCL-7 TPYSPVSTT-A-DAGKSLDEHKRS-DP---PNETTVTYVNPVKELI---NWDAGSYAWEF

*Chitinophaga* sp. *MD30* CDCL-3 QPRSPYTDS-SLHIQASLDEQKRAEDPAQFPG-TVISGINPIREVI---NWNTGAYSWLV

*Chitinophaga* sp. *MD30* CDCL-2 VPRAPQVDS-ADYLRQLHEAQQMSGDPLQPLPVGTLGYVNPIKELI---NWDAGSYSWQF

*Chitinophaga* sp. *MD30* CDCL-6 --------------------EQKRSDP--P---NTIAYVNPIKEWI---NWDVGSYAWDF

*Chitinophaga* sp. *MD30* CDCL-4 ---APYASMDPIGVRQALDQQKLSGDPDFPGRPGSIGYVNPVRDKVFDLDGATSSYSIFF

*Chitinophaga* sp. *MD30* CDCL-5 ---LPDSLHPFLDAAAVLAAHKAAGNPEIPGGGISVTPHGP--SFTDVLNGATYNYAELF

.: :* : .* . : . *: .

*Chitinophaga* sp. *MD30* CDCL-1 ESNEHAVYSDYEDQIYPGALIKGNSVESFDFNPVVG-YTPKPISVSVSLPAPASLVAANI

*Chitinophaga* sp. *MD30* CDCL-7 EANEHVVYSDYEDQIYPGALIKGNSVASFDFNPVIG-YTPKPISLSVSLPAPAGLVAGTI

*Chitinophaga* sp. *MD30* CDCL-3 EANEHVVYTDYEDQIFPGALIKGNSVENFDFNPVIG-YTPKPISVSVSLPAPAGKVAATI

*Chitinophaga* sp. *MD30* CDCL-2 EANEHVVYSDYEDQIYPGALIKGNSVANFAFNPVIG-YTPKPIKVSVSLPAPASKVAATI

*Chitinophaga* sp. *MD30* CDCL-6 ESNEHVVYSDYEDQIYPGALIKGNSIADFSFNPVVG-YTPKPIKVSVSLPAPASKVSATI

*Chitinophaga* sp. *MD30* CDCL-4 ESNEHAVYDDYADQIYPGALINGKSVETFEFNPVIGNYTPKPITVSVSLPAQPSAVAGTI

*Chitinophaga* sp. *MD30* CDCL-5 DANEFVVTTGNTNVIYPGSIIKGSSIEDFGLIPILGY--GRPVTVSVSIPTSPLKVSKTL

::**..* . : *:**::*:*.*: * : *::* :*:.:***:*: . *: .:

*Chitinophaga* sp. *MD30* CDCL-1 AVPSLTATTQMVNNVLLNSEFSQSGFSKYNLNIKEFTYYDELKEFFATSKNTNAIFFNSS

*Chitinophaga* sp. *MD30* CDCL-7 NVPSLSATTQLVNHVLLNSTFAQGGFSKYNLNIKQFTYYDELKEFFASSKNTNLIFFNSG

*Chitinophaga* sp. *MD30* CDCL-3 DIPSLSATTRQVNNVLLNSQFAQLGFAKYNLNIKEFTYYDELKELFATGKNTNLIFFNSS

*Chitinophaga* sp. *MD30* CDCL-2 NAPSYTSMVQTVNNVLLNSALSGGGASKFNFNTKEFTYYDELKEFFASSVNTNAIFFNTS

*Chitinophaga* sp. *MD30* CDCL-6 NSPSYSSMVQTVNNVLLNSPLAGGGFSKFNFNTKEFSYYDELKEYFASGVNTNAIFFNTS

*Chitinophaga* sp. *MD30* CDCL-4 PLPSRTATDRLVNNILLGGSVPNGGFSKYNLNIKEFSFYDELKEFFATGVNTNAIFFNKS

*Chitinophaga* sp. *MD30* CDCL-5 PIATPSGMRQVVRDALTTDFAGSAGFSRFNYQMKAFTYYQELKTLYGYNTKNNLLFVTNS

.: :. : *.. * . * :::* : * *::*:*** :. . :.* :*....

*Chitinophaga* sp. *MD30* CDCL-1 STGSTGIKKISKTTGLMAKFIQKNFTVDMDIPKAGQLIDLNVDAGILGAYSPLYVSSVTY

*Chitinophaga* sp. *MD30* CDCL-7 SSGSTDIKKISRNTGLMAKFIQKNFTVDMDIPKAGQLIDLNVDAGILGAYSPLYVSSVTY

*Chitinophaga* sp. *MD30* CDCL-3 SGTTTNIKKISRSTGLMAKFIQKNFTIDMDIPKAGQLIDLNVDPNIIGAYSPLYVSSVTY

*Chitinophaga* sp. *MD30* CDCL-2 SSGTTNIRKISKRTGLMAKFIQKNFTIDMDIPKAGELIDLNVDQQILGNYAPVYVSSVTY

*Chitinophaga* sp. *MD30* CDCL-6 STGTTNYRKIAKKTGLMAKFIQKNFTLDMDIPKAGELIDLNVDQQILTTYNPTYVSSVTY

*Chitinophaga* sp. *MD30* CDCL-4 TGGTRDVKKIARQTGLMAKFIQKNFTVDMDIPKAGQLLDA-ADVAALGQYSPLYVSSVTY

*Chitinophaga* sp. *MD30* CDCL-5 THIDKNLAQISGKSGIMVKFIQQNFSIDMDIPEAGQLIDPNVDPSVYGGYQPIYISSVIY

: . :*: :*:*.****:**::*****:**:*:* .* * * *:*** *

*Chitinophaga* sp. *MD30* CDCL-1 GRLGIITVESNASFDELKTAFQKAFGILGIVNGSNTLTQAEINIINGADIKVYLVGGQGA

*Chitinophaga* sp. *MD30* CDCL-7 GRLGIITVESNASFDELKTAFQKAFGILGVVNSSNTLTQAEINIINGANIKVYLVGGQGA

*Chitinophaga* sp. *MD30* CDCL-3 GRLGIITVESDAGFDELKKAFEKAFGILGIVNGSNTLTQEEINTINSADIKIYLVGGLGA

*Chitinophaga* sp. *MD30* CDCL-2 GRLGIITVESDADFEQLNQAFKKAFGILGIVNGTNTLTQDEINLINAADIKVYLVGGDGA

*Chitinophaga* sp. *MD30* CDCL-6 GRLGIISVESDADFEQLNQAFKKAFGILGIVNGTNTLTQDEINLINAADIKVYLIGGEGA

*Chitinophaga* sp. *MD30* CDCL-4 GRLGIIAVESNYSFDKLNTAFKKAFNIIGI-NGSNTLTQEELNVINSADIKVYLVGGNGS

*Chitinophaga* sp. *MD30* CDCL-5 GRMGIMTIESKAEQSKAEATFRKAFNVLGIINGGNSLTSEETALIDSAEIKVSVAGVSGE

**:**:::**. .: : :*.***.::*: *. *:**. * *:.*:**: : * *

*Chitinophaga* sp. *MD30* CDCL-1 MAVQTMSGYQHFMQYLGGGQTFSAQTPGMPIVFSMRYLSDHKPYKAKFQINYGNIEKVYA

*Chitinophaga* sp. *MD30* CDCL-7 MAVQTMNGYQHFMQYLGGGQTFSPQTPGVPISFSMRYLSDHRPYKAQFQINYGPIAKVYT

*Chitinophaga* sp. *MD30* CDCL-3 QAVQTINGYQNFLQYLGGGQTFSAQAPGVPITFNMRYLSDHSAYKAQFQINYGNIEKVYA

*Chitinophaga* sp. *MD30* CDCL-2 KAVQTINGYQNFLQYLGGGQTFTPQTPGVPISFSLRYLSDHSACKAQFQVNYGPISRTYA

*Chitinophaga* sp. *MD30* CDCL-6 KAVQTINGYQNFLQYMGGGQTFSPQAPGVPIAFSMRYLKDHSACKAQFQINYGPISRTYA

*Chitinophaga* sp. *MD30* CDCL-4 QAVATINGFEHFKSYLGGGQTFSADAPGVPITFSLRYLSDHAAYKAKFEINYGSIDKVYA

*Chitinophaga* sp. *MD30* CDCL-5 EAVRLVGGVQGLTALLAKGMTYDAASPGVPIAFKMKDIATDSLIAAPFQVDYGIYDKVYA

** :.* : : :. * *: . :**:** *.:: : . * *:::** :.*:

**Domain 4→**

*Chitinophaga* sp. *MD30* CDCL-1 **RIETAWSDKKE----PFPPP--------YRKFYFYNVFLAFYSDAACTKPMKASNVIKFS**

*Chitinophaga* sp. *MD30* CDCL-7 **RIELR-NYKTEYATSPFGYQ--------YRVGEYADVHIAFYQDANCTMPTRAYNFVQFN**

*Chitinophaga* sp. *MD30* CDCL-3 **RIE--FSNQRNELSFPSAGS--------TRETTIEDIYLAFYQDVNCTKSVKASNVIQFD**

*Chitinophaga* sp. *MD30* CDCL-2 **RFE--YRNQTYPMKWGYAPE-QNGVIVGTMPDLYADVHLAFYQDASCTMPVKAYNFVKYN**

*Chitinophaga* sp. *MD30* CDCL-6 **RFE--YRNQTSPIYWGNAPG-QPGVIVGTMPSTFSDIHLAFYQDASCTMPVKAYNFVKYN**

*Chitinophaga* sp. *MD30* CDCL-4 **RFEYRNVRFLPPVYNDPADEIGEGQTSWSKTARVGDLRIGFYSNSACTQPVAARNFVGFN**

*Chitinophaga* sp. *MD30* CDCL-5 **SLEIENRYQVGTGGAGATYE-------------YGDLYLAFYQWPNKIQPVAAPNFVPFN**

**:* :: :.**. . * *.: :.**

*Chitinophaga* sp. *MD30* CDCL-1 **YNY-TTRGGLNR-PMSDVYTSPAT--V---IDNVQKGTKILLEQDALMEVVISGSAPPDM**

*Chitinophaga* sp. *MD30* CDCL-7 **FEQ-RNRSELNLVPEPPRITEDISNYS---IKNELKGTSVLVGAHVRL------ASYPDY**

*Chitinophaga* sp. *MD30* CDCL-3 **YSV-TLSKNSR---EPGVTTTPVTTTTEKKVKNTAKGTRILLEKDK-----ITYSKTSKG**

*Chitinophaga* sp. *MD30* CDCL-2 **YQLHTLTIEIIPAPVS---GGVTNTYDKGTRQNDQKGTSIYMGNILINSLD---EHVTSK**

*Chitinophaga* sp. *MD30* CDCL-6 **YEVWTQTTQIVPNPFSEGWNGITDRYERSTIRNELKGTSIFLGNALIRSLD---QRVSNQ**

*Chitinophaga* sp. *MD30* CDCL-4 **VE---RSEYYVRSPEGYSYTYPFN----ELRKNELKGNYILLSNELFLSSEV--ESFNNQ**

*Chitinophaga* sp. *MD30* CDCL-5 **YLINTVTENRQYGPNGYHNRSEKN--EDVKVRNSLKNTRQLLQKDARLRYLVKYYD-PNQ**

*** *.. : .**

*Chitinophaga* sp. *MD30* CDCL-1 **PTGTEYQK---TYTLLPGNGYT----V--K**

*Chitinophaga* sp. *MD30* CDCL-7 **DSEQNYEQFFINFKMLPGEGYY----IA-K**

*Chitinophaga* sp. *MD30* CDCL-3 **GRGGSFYSSSYHYVLRSGNGYTTSAPVW-N**

*Chitinophaga* sp. *MD30* CDCL-2 **GVVTERTKIYRTYSLLPGEGYIIVNN---P**

*Chitinophaga* sp. *MD30* CDCL-6 **GVVTESDKLTREYRLLPGEGYIVAPT---R**

*Chitinophaga* sp. *MD30* CDCL-4 **YPGSTLVTRKHTYKLLPGAGYIIAPEVTIR**

*Chitinophaga* sp. *MD30* CDCL-5 **PNNGNQSESIYTYRLKPGPGYIVQP-----**

**: : .* ****
