# Supplementary material for: A Key Motif in the Cholesterol-Dependent Cytolysins Reveals a Large Family of Related Proteins
Source: mBio. 2020 Sep 29;11(5):e02351-20. doi: 10.1128/mBio.02351-20 (PMC7527733; doi:10.1128/mBio.02351-20)
Supplement: TABLE S2 [file mBio.02351-20-st002.docx]

## **Table S2.** X-ray diffraction data collection and refinement statistics

| Resolution range | 20.15 - 2.1 (2.175 - 2.1) |
| --- | --- |
| Space group | *P*4_2_2_1_2 |
| Unit cell (Å) | 121.74 121.74 88.91 |
| Total reflections | 2,512,703 (165,191) |
| Unique reflections | 39,545 (3,893) |
| Multiplicity | 63.5 (42.4) |
| Completeness (%) | 99.97 (100.0) |
| Mean I/sigma(I) | 11.17 (1.43) |
| Wilson B-factor | 45.87 |
| R-pim (%) | 5.9 (41.5) |
| CC1/2 | 0.997 (0.533) |
| CC* | 0.999 (0.834) |
| Reflections used in refinement | 36,543 (3,893) |
| Reflections used for *R*-free | 1988 (196) |
| *R*-work | 0.2021 (0.3655) |
| *R*-free | 0.2466 (0.3834) |
| Number of non-hydrogen atoms | 3793 |
| macromolecules | 3680 |
| ligands | 13 |
| Protein residues | 458 |
| RMS bonds (Å) | 0.015 |
| RMS angles (°) | 1.56 |
| Ramachandran favored (%) | 94.9 |
| Ramachandran outliers (%) | 0.44 |
| Rotamer outliers (%) | 0.24 |
| Clashscore | 1.20 |
| Average B-factor (Å^2^) | 59.8 |
| macromolecules | 60.0 |
| ligands | 57.4 |
| solvent | 51.6 |
